# Additive-assisted regioselective 1,3-dipolar cycloaddition of azomethine ylides with benzylideneacetone

**DOI:** 10.3762/bjoc.10.33

**Published:** 2014-02-07

**Authors:** Chuqin Peng, Jiwei Ren, Jun-An Xiao, Honggang Zhang, Hua Yang, Yiming Luo

**Affiliations:** 1 School of Chemistry and Chemical Engineering, Central South University,Changsha, Hunan 410083, P. R. China

**Keywords:** 1,3-dipolar cycloaddition, azomethine ylide, regioselectivity, spirooxindole

## Abstract

1,3-Dipolar cycloadditions of isatins, benzylamine and benzylideneacetones were studied to prepare a series of novel spiropyrrolidine-oxindoles – 4′-acetyl-3′,5′-diarylspiro[indoline-3,2′-pyrrolidin]-2-ones and 3′-acetyl-4′,5′-diarylspiro[indoline-3,2′-pyrrolidine]-2-ones in good yields of up to 94% and with good regioselectivities. Regioselectivities are reversible by the addition of water or 4-nitrobenzoic acid, respectively. The substituent effects on the regioselectivity were also investigated.

## Introduction

Spirooxindoles are important synthetic targets due to their significant biological activities and their applications for pharmaceutical lead discovery. These compounds are the central skeleton of numerous alkaloids [1–8] and have found wide biological applications, e.g., as potent p53–MDM2 inhibitors [9–15]. Usually, isatin and its derivatives were employed as starting materials to conduct 1,3-dipolar cycloadditions to yield spirooxindole core structures [16–20]. Owing to the ease of preparation, the azomethine ylides generated from isatin with α-amino acids or amines were frequently chosen as important 1,3-dipolar intermediates to react with various dipolarophiles, such as α,β-unsaturated esters [21–25], dienones [26–27], α,β-unsaturated ketones [28–30], unsaturated aryl ketones [31–33] and electron-poor alkenes [34–39].

Among the studied α,β-unsaturated enones for 1,3-dipolar cycloaddition, chalcone derivatives are the most widely used dipolarophiles. Sarrafi and co-workers reported a 1,3-dipolar cycloaddition reaction of isatin, benzylamine and chalcone derivatives [31], and only one single regioisomer was obtained in high yield, in which the benzoyl group was connected to C-3 of the newly-constructed pyrrolidine. However, unsaturated ketones with α-hydrogens such as benzylideneacetone, which have attracted great interest due to their synthetic potential [40–42], have not been exhaustively studied as suitable dipolarophiles for 1,3-dipolar cycloadditions of azomethine ylides to prepare spirooxindoles yet [43]. Therefore, extensive studies on the regioselective 1,3-dipolar cycloaddition of azomethine ylides using simple unsaturated ketones, especially ketones having α-hydrogens, are highly desirable, to enrich the library of spirooxindoles and facilitate their biological investigations.

**Scheme 1:**
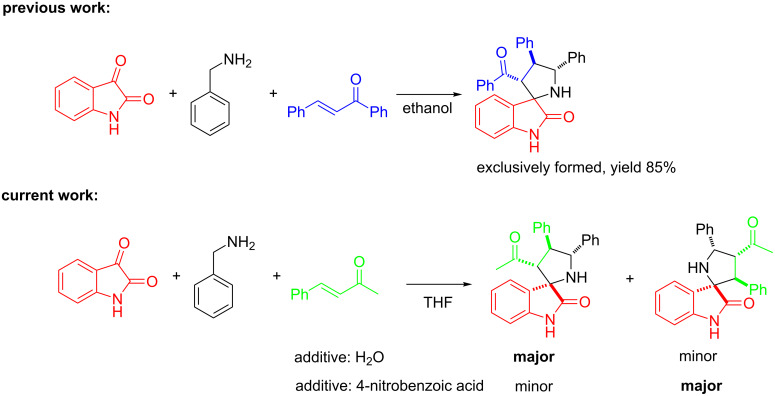
Different regioselectivities in 1,3-dipolar cycloaddition of azomethine ylide.

Our group recently reported an unusual regioselectivity when 3-acetonylideneoxindoles were employed as dipolarophiles to react with azomethine ylides [44]. The structure of the substrate significantly affected the regioselectivity of the 1,3-dipolar cycloaddition, which allowed the formation of 3-acetyl-5-phenyl-pyrrolo(spiro-[2.3′]-1′-benzyloxindole)-spiro-[4.3″]-1″-benzyloxindoles in good regioselectivity. Our continued interest in the regioselective 1,3-dipolar cycloaddition of azomethine ylides prompted us to further investigate the regioselectivity of the 1,3-dipolar cycloaddition using α,β-unsaturated enones. Moreover, we envisioned that the additive might effectively tune the regioselectivity of a 1,3-dipolar cycloaddition of azomethine ylide. Herein, we report a three-component 1,3-dipolar cycloaddition of azomethine ylides, generated in situ from isatin derivatives and benzylamine, with benzylideneacetone derivatives in the presence of various additives. It was found that the addition of water can significantly improve the regioselectivity and yield of this reaction [45–48]. More importantly, the regioselectivity of the 1,3-dipolar cycloaddition of azomethine ylide was reversed by the addition of 4-nitrobenzoic acid, which led to the formation of spirooxindoles with novel substitution patterns (Scheme 1). Accordingly, a series of novel functionalized 3-spiropyrrolidine-oxindoles bearing an acetyl group were prepared via this 1,3-dipolar cycloaddition with up to 94% yield. To the best of our knowledge, the reversal of the regioselectivity in the 1,3-dipolar cycloaddition of azomethine ylide induced by the additive is reported for the first time.

## Results and Discussion

Initially, a three-component reaction of isatin (**1a**), benzylamine (**2**) and benzylideneacetone (**3a**) was conducted in ethanol at room temperature (Table 1). It smoothly went until completion. Interestingly, the two regioisomers **4a** and **5a** were obtained with modest yield and poor regioisomeric ratio (Table 1, entry 1), which is quite different from the reaction of chalcone. Generally, only a single regioisomer 4′,5′-diarylspiro(indoline-3,2′-pyrrolidin)-2-one was formed when using chalcone or dienone as dipolarophiles [20,31]. Presumably, this might be attributed to the electronic and steric effects of the acetyl group. Therefore, reaction conditions including various solvents and additives (Table 1, entries 2–9) were screened to improve the regioselectivity in this reaction. It turned out that the addition of triethylamine or the removal of water by using molecular sieves slightly decreased both the yield and the regioisomeric ratio (Table 1, entry 2 and entry 3). However, the incorporation of 4-nitrobenzoic acid can favor the formation of regioisomer **5a** with a regioisomeric ratio of 50:50 (Table 1, entry 4). Interestingly, the employment of water as an additive resulted in a significant improvement of the regioisomeric ratio and a slightly decreased yield (Table 1, entry 6). Encouraged by this result, we studied the effect of the amount of water on the regioselectivity. When the amount of water was increased to 5.0 equiv, the ratio of **4a**/**5a** could be improved to 83:17 (Table 1, entry 5). Meanwhile, as the addition of water was further increased (from 5.0 equiv to 1:1, Table 1, entry 6 and entry 7), the regioisomeric ratio dropped slightly and leveled off. The use of water as a solvent led to a poor yield with eroded regioselectivity (Table 1, entry 8). Various solvents with 5.0 equiv of water as an additive were subsequently investigated. The best regioselectivity was obtained with THF as a solvent (Table 1, entries 9–11). The amount of isatin is also important for the yield, and the yield was improved to 88% when 1.5 equiv of isatin were used (Table 1, entry 12). This might be due to the instability of the corresponding azomethine ylides, and an excess of isatin and benzylamine was therefore needed.

**Table 1:** 1,3-Dipolar cycloaddition reaction of isatin (**1a**) and benzylamine (**2**) with benzylideneacetone (**3a**)^a^.

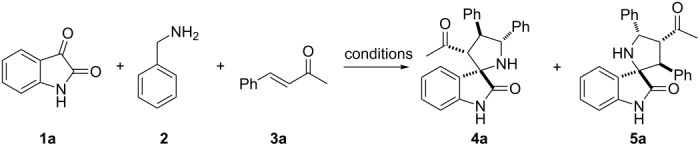					

Entry	Solvent^b^	Additive	Time (h)	Yield (%)^c^	Regioisomeric ratio (**4a**/**5a**)^d^

1	EtOH	–	48	72	75:25
2	EtOH	Et_3_N (0.2 equiv)	72	69	78:22
3	EtOH	4 Å MS	48	59	76:24
4	EtOH	4-NO_2_PhCO_2_H (0.2 equiv)	24	33	50:50
5	EtOH	H_2_O (5.0 equiv)	24	54	83:17
6	EtOH	H_2_O (20 equiv)	24	52	76:24
7	EtOH	EtOH:H_2_O (1:1)	24	50	75:25
8	H_2_O	–	72	23	68:32
9	DMF	H_2_O (5.0 equiv)	18	78	84:16
10	CH_3_CN	H_2_O (5.0 equiv)	48	56	67:33
11	THF	H_2_O (5.0 equiv)	24	71	86:14
**12**^e^	**THF**	**H_2_O (5.0 equiv)**	**24**	**88**	**86:14**

^a^Unless otherwise noted, all reactions were carried out in sealed reaction vials at rt with isatin (**1a**, 0.50 mmol), benzylamine (**2**, 1.0 mmol), benzylideneacetone (**3a**, 0.75 mmol), and additives in solvent (5.0 mL). ^b^Anhydrous solvent was used. ^c^Combined yield of isolated **4a** and **5a**. ^d^The regioisomeric ratio was determined by the isolated yields of **4a** and **5a**. ^e^The ratio of **1a**/**2**/**3a** is 1.5:2:1.

As shown in Table 1 (entry 4), the addition of acid facilitated the formation of regioisomer **5a**, which prompted us to further investigate the effects of acid additives. We anticipated that the acid additives might lead to the preferably formation of regioisomer **5a**, which would provide us an efficient pathway to prepare the spirooxindoles with this novel substitution pattern. Thus, the acid additives were examined and the optimization results are listed in Table 2. To our delight, the addition of 4-nitrobenzoic acid reversed the regioselectivity of this reaction, and the ratio of **5a**/**4a** was increased from 50:50 to 70:30 with an improved yield (90%) when the amount of 4-nitrobenzoic acid increased from 0.2 equiv to 2.0 equiv (Table 2, entries 1–5). Presumably, this might be attributed to the acid, which accelerates the formation of azomethine ylide. However, a large excess of acid (10 equiv) has a detrimental effect on the reaction, and the yield of **4a** and **5a** dropped tremendously to 46% (Table 2, entry 6 and entry 7). As a result, 2.0 equiv of 4-nitrobenzoic acid proved to give superior results. Various acid additives were also evaluated. Unfortunately, the corresponding azomethine ylides were not formed as indicated by TLC, and cyclization was not observed with 2.0 equiv *p*-TSA and TFA (Table 2, entry 8 and entry 9). Both the benzoic acid and acetic acid slightly improved the formation of regioisomer **5a** (Table 2, entry 10 and entry 11). Additionally, a cycloaddition product was not observed with acetic acid as a solvent (Table 2, entry 12).

**Table 2:** 1,3-Dipolar cycloaddition reaction of isatin (**1a**) and benzylamine (**2**) with benzylideneacetone (**3a**) and acid additives^a^.

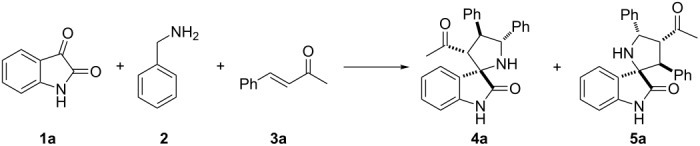					

Entry	Additive	Solvent^b^	Time (h)	Yield (%)^c^	Regioisomeric ratio (**4a**/**5a**)^d^

1	4-NO_2_PhCO_2_H (0.2 equiv)	THF	12	33	50:50
2	4-NO_2_PhCO_2_H (0.5 equiv)	THF	12	69	42:58
3	4-NO_2_PhCO_2_H (1.0 equiv)	THF	12	62	32:68
4	4-NO_2_PhCO_2_H (1.5 equiv)	THF	12	79	31:69
**5**	**4-NO_2_PhCO_2_H (2.0 equiv)**	**THF**	**12**	**90**	**30:70**
6	4-NO_2_PhCO_2_H (5.0 equiv)	THF	12	75	30:70
7	4-NO_2_PhCO_2_H (10.0 equiv)	THF	12	46	37:63
8	*p*-TSA (2.0 equiv)	THF	48	trace	
9	TFA (2.0 equiv)	THF	48	trace	
10	PhCO_2_H (2.0 equiv)	THF	12	75	54:46
11	AcOH (2.0 equiv)	THF	12	85	69:31
12	–	AcOH	48	trace	

^a^Unless otherwise noted, all reactions were carried out in sealed reaction vials with isatin (**1a**, 0.75 mmol), benzylamine (**2**, 1.0 mmol), benzylideneacetone (**3a**, 0.50 mmol) and additives in solvent (5.0 mL) at rt. ^b^Anhydrous solvent was used. ^c^Combined yield of isolated **4a** and **5a**. ^d^The regioisomeric ratio was determined by the isolated yields of **4a** and **5a**.

**Scheme 2:**
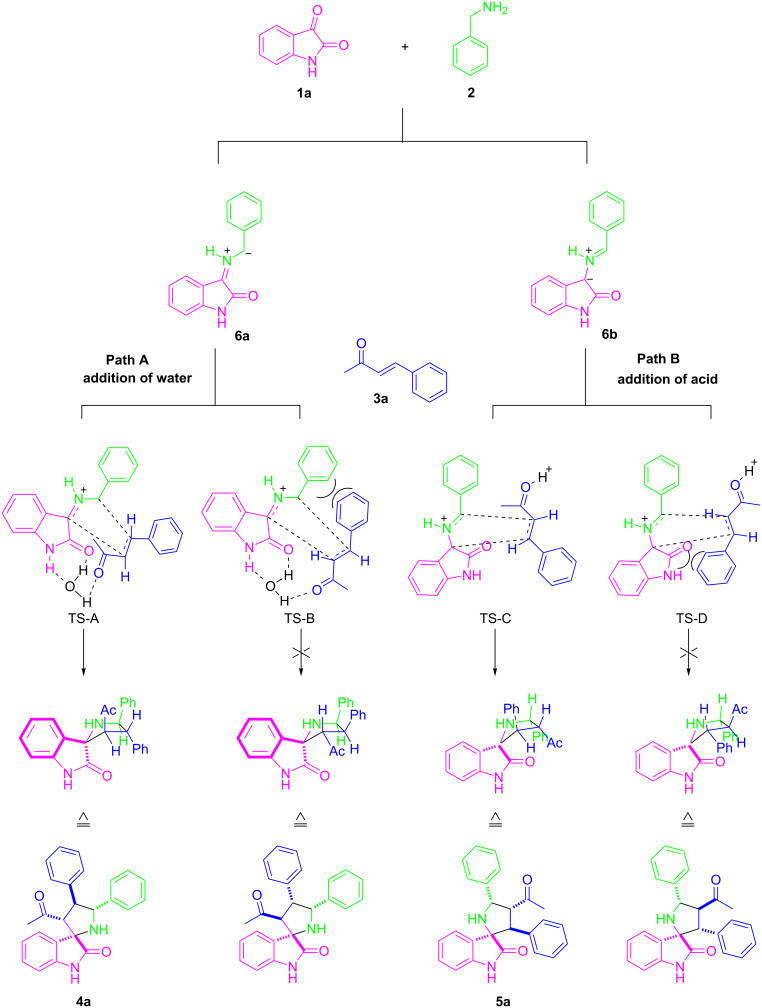
Plausible pathways for the formation of different regioisomers.

A plausible mechanism for the regioselectivity in this transformation is proposed in Scheme 2. The azomethine ylides generated from the reaction of isatin with benzylamine has two potential nucleophilic carbons (**6a** and **6b**) [34], each of which could add to the electron-deficient β-carbon of benzylideneacetone during the cycloaddition leading to two regioisomers [31]. In the presence of water, transition state **A** is favored due to the formation of an intermolecular hydrogen bonding between water and two carbonyl groups in the reaction substrates, while transition state **B** suffers from severe steric repulsion [45–48]. Presumably, the addition of 4-nitrobenzoic acid might facilitate the formation of dipole **6b**. Similarly, the less sterically hindered transition state **C** leads to **5a** as the major product. Further research work on the elaboration of the detailed mechanism is still underway and will be published in due course.

**Figure 1:**
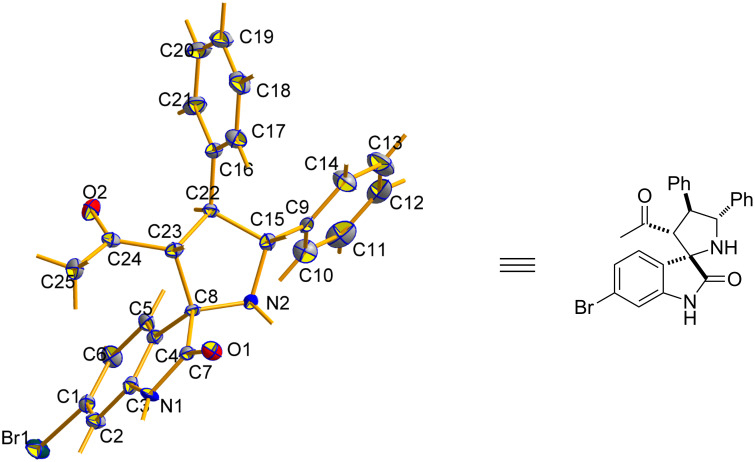
ORTEP diagram of **4e**.

**Figure 2:**
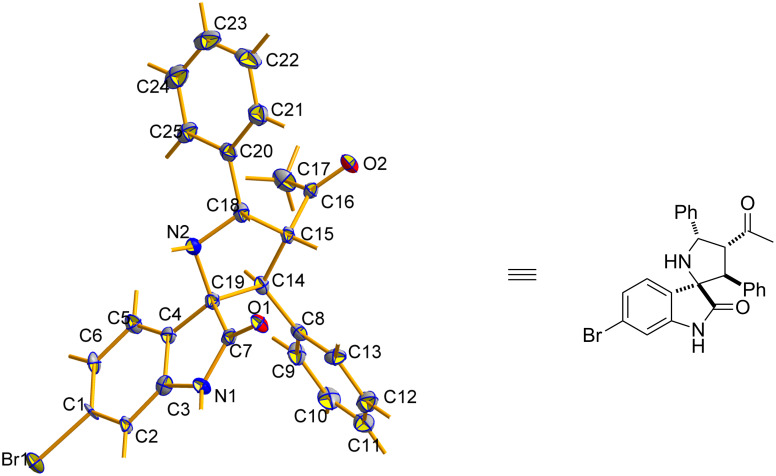
ORTEP diagram of **5e**.

Having established the optimal protocol for this reaction, we next examined the scope of this method with regard to α,β-unsaturated ketones and azomethine ylides. With the aim of applying this additive-assisted regioselective 1,3-dipolar cycloaddition to prepare two regioisomers in high yields, we tested two reaction conditions (conditions A: 5.0 equiv H_2_O as an additive; conditions B: 2.0 equiv 4-NO_2_PhCOOH as additive) for all substrates. As shown in Table 3, the reactions between benzylideneacetone with the azomethine ylides derived from isatin **1a–e** and benzylamine (**2**) proceeded smoothly to furnish the desired products with good yields. The opposite regioselectivities were also observed by using water and 4-nitrobenzoic acid as additives, respectively (Table 3, entries 1–5). The substituents on the phenyl ring of isatin exert a mild influence on the regioselectivities, resulting in slightly lowered yields and regioseletivities (Table 3, entries 2–5). Next, benzylideneacetone derivatives **3a–g** were employed to react with the azomethine ylide derived from isatin (**1a**) and benzylamine (**2**). It was found that the electronic nature of the substituent and its position on the benzylideneacetone aromatic ring significantly influenced the regioisomeric ratio. In general, the regioisomeric ratio with water as an additive is comparatively higher for the substrates in which the phenyl rings of enones were substituted by electron-donating groups (Table 3, entries 6, 10 and 11). When the hydroxy group was introduced to the *para*-position on the phenyl ring of enone, the best regioisomeric ratio was obtained and only one single regioisomer **4k** was isolated (Table 3, entry 11). Surprisingly, the addition of 4-nitrobenzoic acid only slightly facilitated the formation of regioisomers **5h–5j** (Table 3, entries 8–11) and did not yield the reversed regioselectivities. Notably, the regioisomer **5k** was present in trace amounts, even after the addition of 4-nitrobenzoic acid. Finally, the structures and relative configurations of the cycloadducts **4e** and **5e** were unequivocally determined by an X-ray crystallographic analysis of a single crystal (Figure 1 and Figure 2).

**Table 3:** 1,3-Dipolar cycloaddition reaction of isatin derivatives **1a–e** and benzylamine (**2**) with benzylideneacetone derivatives **3a–g**^a^.

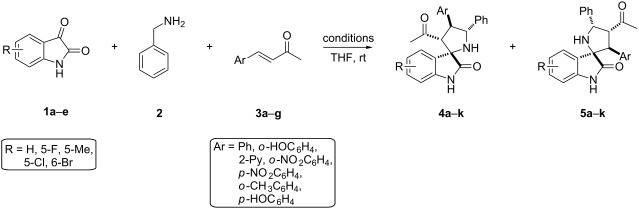						

Entry	R	Ar	Product	Conditions (A or B)^b^	Yield (%)^c^	Regioisomeric ratio (**4a–l**/**5a–l**)^d^

1	H	Ph	**4a** + **5a**	A	88	86:14
				B	90	30:70
2	5-F	Ph	**4b** + **5b**	A	79	74:26
				B	92	38:62
3	5-Me	Ph	**4c** + **5c**	A	88	68:32
				B	89	31:69
4	5-Cl	Ph	**4d** + **5d**	A	69	73:27
				B	67	32:68
5	6-Br	Ph	**4e** + **5e**	A	77	80:20
				B	80	24:76
6	H	*o*-OHC_6_H_4_	**4f** + **5f**	A	86	85:15
				B	80	11:89
7	H	2-Py	**4g** + **5g**	A	90	81:19^e^
				B	84	40:60^e^
8	H	*o*-NO_2_C_6_H_4_	**4h** + **5h**	A	92	67:33
				B	85	55:45
9	H	*p*-NO_2_C_6_H_4_	**4i** + **5i**	A	93	70:30
				B	84	58:42
10	H	*o*-CH_3_C_6_H_4_	**4j** + **5j**	A	92	78:22
				B	93	60:40
11	H	*p*-OHC_6_H_4_	**4k** + **5k**	A	94	97:3^e^
				B	82	99:1^e^

^a^Unless otherwise noted, all reactions were carried out in sealed reaction vials at rt with isatin derivatives **1a–e** (0.75 mmol), benzylamine (**2**, 1.0 mmol), benzylideneacetone derivatives **3a–g** (0.50 mmol), and additives in THF (5.0 mL) for 48 h. ^b^Conditions A: 5.0 equiv H_2_O (2.5 mmol) as additive; conditions B: 2.0 equiv 4-NO_2_PhCOOH (1.0 mmol) as additive. ^c^Combined yield of isolated **4a–k** and **5a–k**. ^d^The regioisomeric ratio was determined by the isolated yields of **4a–k** and **5a-k**. ^e^The regioisomeric ratio was determined by ^1^H NMR of the crude mixture.

## Conclusion

In summary, we herein described an additive-assisted regioselective 1,3-dipolar cycloaddition reaction of azomethine ylide to synthesize novel functionalized spirooxindoles in good to excellent chemical yields with good regioselectivities. Furthermore, the regioselectivity can be conveniently tuned and reversed by simply adding water or 4-nitrobenzoic acid, which provides a facile approach to access a wide range of spirooxindole ring systems with novel substitution patterns.

## Supporting Information

File 1 Experimental procedures, characterization data and copies of ^1^H and ^13^C NMR spectra.
