# Supplementary material for: Additive-assisted regioselective 1,3-dipolar cycloaddition of azomethine ylides with benzylideneacetone
Source: Beilstein J Org Chem. 2014 Feb 7;10:352–60. doi: 10.3762/bjoc.10.33 (PMC3943969; doi:10.3762/bjoc.10.33)
Supplement: File 1 — Experimental procedures, characterization data and copies of 1H and 13C NMR spectra. [file Beilstein_J_Org_Chem-10-352-s001.pdf]

Supporting Information

for

Additive-assisted regioselective 1,3-dipolar cycloaddition of azomethine ylides with benzylideneacetone

Chuqin Peng, Jiwei Ren, Jun-An Xiao, Honggang Zhang, Hua Yang*[§], Yiming Luo*[¶]

Address: School of Chemistry and Chemical Engineering, Central South University,Changsha, Hunan 410083, P. R. China

Email:Yiming Luo - ymluo@csu.edu.cn; Hua Yang - hyangchem@csu.edu.cn

*Corresponding author

§Corresponding author Tel.: +86-731-88830833

¶Corresponding author Tel.: +86-731-88864409

Experimental procedures, characterization data and copies of ^{1}H and ^{13}C NMR spectra

Contents

General information S2

Typical procedure for the synthesis of spirooxindole derivatives S2

Characterization data for spiropyrrolidine oxindoles S3

Copies of NMR spectra for spiropyrrolidine oxindoles..S14

General information

^{1}H NMR spectra were recorded at 400 MHz. The chemical shifts were recorded in ppm relative to tetramethylsilane and with the solvent resonance as the internal standard. Data were reported as follows: chemical shift, multiplicity (s = singlet, d = doublet, t = triplet, q = quartet, m = multiplet), coupling constants (Hz), integration. ^{13}C NMR data were collected at 100 MHz with complete proton decoupling. Chemical shifts are reported in ppm from tetramethylsilane with the solvent resonance as internal standard. Mass spectra (MS) were measured by 3200 Q TRAP LC-MS/MS utilizing electrospray ionization (ESI) and acetonitrile was used to dissolve the sample. All chemicals were obtained from commercial sources and used without further purification. Column chromatography was carried out on silica gel (300–400 mesh). Thin-layer chromatography (TLC) was performed on TLC silica gel 60 F254 plates. Benzylideneacetone derivatives **3a–g** were prepared according to literature procedure [1].

Typical procedure for the synthesis of spirooxindole derivatives

A mixture of isatin (**1a**, 110.3 mg, 0.75 mmol, 1.5 equiv), benzylamine (**2**, 107.1 mg, 1.0 mmol, 2.0 equiv), benzylideneacetone (**3a**, 73.1 mg, 0.5 mmol, 1.0 equiv) and additives (Conditions A: H_2O, 45 mg, 5.0 equiv; Conditions B: 4-nitrobenzoic acid, 167.1 mg, 2.0 equiv) in THF (5.0 mL) was stirred at rt for the indicated time. After completion of the reaction as monitored by TLC, Conditions A: the solvent was removed in vacuo. Conditions B: saturated $NaHCO_3$ solution was added, the organic phase was separated and the aqueous layer was extracted with CH_2Cl_2. The combined organic phases were dried over Na_2SO_4, concentrated under reduced pressure and the

crude mixture was purified via flash silica gel chromatography (eluent: dichloromethane/ethyl acetate 97:3 to 95:5) to give cycloadducts **4a**, white solid, conditions A: 144.5 mg, yield 76%; conditions B: 45.8 mg, yield 27%; **5a**, white solid, conditions A: 23.5 mg, yield 12%; conditions B: 120.3 mg, yield 63%.

Characterization data for spiropyrrolidine oxindoles

Crystal data for 4e and 5e

Crystal data for **4e**: formula: $C_{25}H_{21}Br_1N_2O_2$, unit cell parameters: *a* 7.382(10), *b* 9.93110(10), *c* 16.9977(3), *P*1. Crystallographic data for this structure have been deposited at the Cambridge Crystallographic Data Centre and allocated deposition number CCDC 967232.

Crystal data for **5e**: formula: $C_{25}H_{21}Br_1N_2O_2$, unit cell parameters: *a* 10.3391(7), *b* 10.6015(6), *c* 22.2259(16), *P*21/*c*. Crystallographic data for this structure have been deposited at the Cambridge Crystallographic Data Centre and allocated deposition number CCDC 967233.

Spectral characterization data for spiropyrrolidine oxindoles

3'-Acetyl-4',5'-diphenylspiro[indoline-3,2'-pyrrolidin]-2-one (4a)

White solid (conditions A: 144.5 mg, yield 76%; conditions B: 45.8 mg, yield 27%); m.p. 170-172°C; IR (KBr) ν 3332, 3030, 1712, 1619, 1471, 1188, 753, 700 cm^{-1}; ^{1}H NMR (400 MHz, $CDCl_3$) δ 8.40 (*br* s, 1H), 6.93-7.34 (m, 15H), 4.97-5.02 (m, 1H), 3.96-4.03 (m, 2H), 1.57 (s, 3H); ^{13}C NMR (100 MHz, $CDCl_3$) δ 204.4, 182.9, 140.4, 140.3, 138.7, 130.0, 129.7, 128.6, 128.4, 128.3, 127.7, 127.0, 126.0, 123.6, 110.3, 68.3, 67.9, 55.1, 30.8, 25.4; HRMS (TOF-ES+) m/z: $[M+Na]^+$ calcd for $C_{25}H_{22}N_2O_2Na$ 405.1576, found 405.1598.

4'-Acetyl-3',5'-diphenylspiro[indoline-3,2'-pyrrolidin]-2-one (**5a**)

White solid (conditions A: 23.5 mg, yield 12%; conditions B: 120.3 mg, yield 63%); m.p. 205-206°C; IR (KBr) *v* 3391, 3061, 1709, 1620, 1471, 1187, 753, 699 cm^{-1}; ^{1}H NMR (400 MHz, $CDCl_3$) δ 7.70-7.72 (m, 1H), 7.54 (d, *J* = 7.2 Hz, 2H), 7.35 (t, *J* = 7.2 Hz, 2H), 6.99-7.29 (m, 9H), 6.59-6.61 (m, 1H), 5.65 (d, *J* = 10.4 Hz, 1H), 4.74 (t, *J* = 11.2 Hz, 1H), 4.32 (d, *J* = 11.6 Hz, 1H), 2.58 (s, 1H), 1.65 (s, 3H); ^{13}C NMR (100 MHz, $CDCl_3$) δ 205.8, 180.2, 147.3, 142.9, 141.0, 140.4, 129.7, 128.9, 128.8, 128.5, 128.3, 124.0, 123.39, 123.33, 109.9, 72.4, 61.0, 58.9, 54.6, 31.2; HRMS (TOF-ES+) m/z: $[M+H]^+$ calcd for $C_{25}H_{23}N_2O_3$ 383.1760, found 383.1752.

3'-Acetyl-5-fluoro-4',5'-diphenylspiro[indoline-3,2'-pyrrolidin]-2-one (**4b**)

White solid (conditions A: 117.0 mg, yield 59%; conditions B: 69.9 mg, yield 35%); m.p. 122-123°C; IR (KBr) *v* 3426, 3062, 3031, 2923, 1714, 1630, 1484, 1186, 816, 755, 699 cm^{-1}; ^{1}H NMR (400 MHz, DMSO-d_6) δ 10.62 (*br* s, 1H), 7.17-7.24 (m, 10H), 7.07 (d, *J* = 8.0 Hz, 2H), 6.84-6.87 (m, 1H), 4.79 (dd, *J* = 10.0, 5.2 Hz, 1H), 4.04 (d, *J* = 5.2 Hz, 1H), 3.85 (d, *J* = 10.8 Hz, 1H), 3.76 (d, *J* = 10.4 Hz, 1H), 1.53 (s, 3H); ^{13}C NMR (100 MHz, DMSO-d_6) δ 204.9, 182.3, 159.7, 157.3, 141.7, 139.6, 138.5, 133.1, 128.8, 128.7, 128.4, 127.8, 127.7, 127.2, 116.1, 115.8, 114.0, 113.7, 110.9, 110.8, 67.9, 67.67, 67.66, 67.61, 54.8, 30.8; HRMS (TOF-ES+) m/z: $[M+Na]^+$ calcd for $C_{25}H_{21}N_2O_2NaF$ 423.1485, found 423.1480.

4'-Acetyl-5-fluoro-3',5'-diphenylspiro[indoline-3,2'-pyrrolidin]-2-one (**5b**)

White solid (conditions A: 41.1 mg, yield 21%; conditions B: 114.1 mg, yield 57%); m.p. 243-245°C; IR (KBr) *v* 3372, 3331, 1715, 1637, 1490, 1171, 809, 750, 700 cm^{-1}; ^1H NMR (400 MHz, DMSO-d_6) δ 9.90 (s, 1H), 7.55-7.60 (m, 3H), 7.34 (t, J = 7.6 Hz 2H), 7.25 (t, J = 7.6 Hz, 1H), 6.95-7.12 (m, 6H), 6.55 (dd, J = 8.4, 4.4 Hz, 1H), 5.46 (dd, J = 10.6, 5.8 Hz, 1H), 4.80 (t, J = 11.6 Hz, 1H), 4.14-4.18 (m, 2H), 1.65 (s, 3H); ^{13}C NMR (100 MHz, DMSO-d_6) δ 206.6, 180.8, 160.0, 157.6, 143.4, 138.8, 136.0, 133.1, 133.0, 129.0, 128.4, 128.3, 127.8, 127.6, 115.5, 115.3, 112.3, 112.1, 110.3, 110.2, 72.8, 60.2, 58.3, 54.2, 31.5; HRMS (TOF-ES+) m/z: $[M+Na]^+$ calcd for $C_{25}H_{21}N_2O_2NaF$ 423.1485, found 423.1503.

3'-Acetyl-5-methyl-4',5'-diphenylspiro[indoline-3,2'-pyrrolidin]-2-one (**4c**)

White solid (conditions A: 118.5 mg, yield 60%; conditions B: 54.7 mg, yield 28%); m.p. 183-184°C; IR (KBr) *v* 3328, 3185, 3030, 2918, 1706, 1624, 1489, 1204, 819, 754, 699 cm^{-1}; ^1H NMR (400 MHz, DMSO-d_6) δ 10.48 (s, 1H), 7.04-7.28 (m, 11H), 6.75 (d, J = 8.4 Hz, 1H), 4.80 (dd, J = 10.0, 5.2 Hz, 1H), 3.71-3.87 (m, 3H), 2.28 (s, 3H), 1.47 (s, 3H); ^{13}C NMR (100 MHz, DMSO-d_6) δ 204.7, 182.4, 142.0, 140.0, 139.9, 131.2, 131.1, 129.8, 128.8, 128.7, 128.5, 127.8, 127.6, 127.2, 126.8, 109.8, 67.9, 67.7, 67.4, 55.0, 30.7, 21.3; HRMS (TOF-ES+) m/z: $[M+Na]^+$ calcd for $C_{26}H_{24}N_2O_2Na$ 419.1735, found 419.1742.

4'-Acetyl-5-methyl-3',5'-diphenylspiro[indoline-3,2'-pyrrolidin]-2-one (**5c**)

White solid (conditions A: 55.8 mg, yield 28%; conditions B: 121.6 mg, yield 61%); m.p. 186-187°C; IR (KBr) *v* 3342, 3316, 3027, 2917, 2870, 1711, 1625, 1491, 1162,

818, 757, 700, 699 cm^{-1}; ^{1}H NMR (400 MHz, DMSO-*d*$_6$) *δ* 9.77 (s, 1H), 7.58 (d, *J* = 7.2 Hz, 2H), 7.50 (s, 1H), 7.34 (t, *J* = 7.2 Hz, 2H), 7.27 (t, *J* = 7.2 Hz, 1H), 6.94-7.10 (m, 6H), 6.46 (d, *J* = 7.6 Hz, 1H), 5.48 (dd, *J* = 10.4, 5.6 Hz, 1H), 4.79 (t, *J* = 11.2 Hz, 1H), 4.12 (d, *J* = 12.0 Hz, 1H), 4.03 (d, *J* = 6.0 Hz, 1H), 2.37 (s, 3H), 1.65 (s, 3H); ^{13}C NMR (100 MHz, DMSO-*d*$_6$) *δ* 206.8, 180.7, 143.5, 140.3, 136.4, 131.0, 130.9, 129.4, 128.9, 128.3, 172.7, 127.4, 124.9, 109.3, 72.4, 60.2, 58.5, 54.2, 31.6, 21.3; HRMS (TOF-ES+) m/z: [M+Na]$^+$ calcd for $C_{26}H_{24}N_2O_2Na$ 419.1735, found 419.1736.

3'-Acetyl-5-chloro-4',5'-diphenylspiro[indoline-3,2'-pyrrolidin]-2-one (**4d**)
White powder (conditions A: 104.8 mg, yield 50%; conditions B: 38.8 mg, yield 21%); m.p 130-132°C; IR (KBr) *v* 3420, 3031, 2924, 1713, 1619, 1474, 1183, 817, 748, 700 cm^{-1}; ^{1}H NMR (400 MHz, $CDCl_3$) *δ* 8.61 (s, 1H), 7.19-7.35 (m, 12H), 6.88 (d, *J* = 8.4 Hz, 1H), 4.95 (d, *J* = 10.0 Hz, 1H), 4.01 (d, *J* = 10.8 Hz, 1H), 3.90 (t, *J* = 10.4 Hz, 1H), 2.55 (s, 1H), 1.62 (s, 1H); ^{13}C NMR (100 MHz, $CDCl_3$) *δ* 203.9, 182.5, 140.0, 138.7, 138.3, 132.0, 129.6, 128.9, 128.6, 128.39, 128.34, 127.8, 127.1, 126.9, 126.6, 111.5, 68.2, 67.8, 67.6, 55.1, 30.8; HRMS (TOF-ES+) m/z: [M+H]$^+$ calcd for $C_{25}H_{22}N_2O_2Cl$ 417.1370, found 417.1359.

4'-Acetyl-5-chloro-3',5'-diphenylspiro[indoline-3,2'-pyrrolidin]-2-one (**5d**)
White solid (conditions A: 38.8 mg, yield 19%; conditions B: 94.8 mg, yield 46%); m.p. 240-241°C; IR (KBr) *v* 3442, 3353, 3282, 1714, 1622, 1480, 1176, 815, 749, 701 cm^{-1}; ^{1}H NMR (400 MHz, DMSO-*d6*) *δ* 10.01 (s, 1H), 7.71 (d, *J* = 2.0 Hz, 1H), 7.58 (d, *J* = 7.2 Hz, 1H), 6.97-7.36 (m, 9H), 6.57 (d, *J* = 8.4, 1H), 5.47 (dd, *J* = 16.8, 6.0

Hz, 1H), 4.77 (t, *J* = 11.2 Hz, 1H), 4.14-4.20 (m, 2H), 1.63 (s, 3H). ^{13}C NMR (100 MHz, DMSO-d_6) δ 206.5, 180.5, 143.3, 141.6, 135.9, 133.2, 129.0, 128.9, 128.4, 128.28, 128.24, 127.7, 127.6, 126.2, 124.5, 110.9, 72.5, 60.2, 58.3, 54.2, 31.4; HRMS (TOF-ES+) m/z: $[M+Na]^+$ calcd for $C_{25}H_{21}N_2O_2NaCl$ 439.1168, found 439.1189.

3'-Acetyl-6-bromo-4',5'-diphenylspiro[indoline-3,2'-pyrrolidin]-2-one (**4e**)

White solid (conditions A: 141.7 mg, yield 62%; conditions B: 88.3 mg, yield 19%); m.p. 176-178°C; IR (KBr) *v* 3325, 3182, 3029, 1708, 1612, 1452, 1325, 699 cm^{-1}; ^{1}H NMR (400 MHz, DMSO-d_6) δ 10.74 (s, 1H), 7.63-7.17 (m, 12H), 7.016-7.020 (m, 1H), 4.80 (dd, *J* = 10.0, 4.8 Hz, 1H), 4.03 (d, *J* = 4.8 Hz, 1H), 3.83 (d, *J* = 10.8 Hz, 1H), 3.74 (t, *J* = 10.0 Hz, 1H), 1.53 (s, 3H); ^{13}C NMR (100 MHz, DMSO-d_6) δ 204.8, 182.2, 144.0, 141.8, 139.6, 130.6, 128.9, 128.7, 128.5, 128.1, 127.8, 127.6, 127.2, 125.0, 122.1, 112.9, 67.8, 67.4, 55.4, 55.0, 30.9; HRMS (TOF-ES+) m/z: $[M+H]^+$ calcd for $C_{25}H_{22}N_2O_2Br$ 461.0865, found 461.0861.

4'-Acetyl-6-bromo-3',5'-diphenylspiro[indoline-3,2'-pyrrolidin]-2-one (**5e**)

White solid (conditions A: 35.4 mg, yield 15%; conditions B: 139.8 mg, yield 61%); m.p. 232-234°C; IR (KBr) *v* 3390, 3193, 3030, 1709, 1610, 1452, 1326, 700 cm^{-1}; ^{1}H NMR (400 MHz, DMSO-d_6) δ 10.02 (s, 1H), 7.64 (d, *J* = 8.0 Hz, 1H), 7.57 (d, *J* = 7.6 Hz, 2H), 7.25-7.35 (m, 4H), 7.12-7.13 (m, 3H), 6.96-6.98 (m, 2H), 6.70-6.71 (m, 1H), 5.46 (dd, *J* = 10.8, 6.0 Hz, 1H), 4.79 (t, *J* = 11.2 Hz, 1H), 4.12-4.18 (m, 2H), 1.65 (s, 3H); ^{13}C NMR (100 MHz, DMSO-d_6) δ 206.5, 180.6, 144.4, 143.4, 136.0, 130.4, 128.9, 128.4, 128.3, 127.8, 127.6, 126.4, 124.8, 121.6, 112.3, 72.2, 60.1, 58.2, 54.0,

31.5; HRMS (TOF-ES+) m/z: $[M+Na]^+$ calcd for $C_{25}H_{21}N_2O_2NaBr$ 483.0684, found 483.0681.

3'-Acetyl-4'-(2-hydroxyphenyl)-5'-phenylspiro[indoline-3,2'-pyrrolidin]-2-one (4f)

White solid (conditions A: 145.5 mg, yield 73%; conditions B: 17.5 mg, yield 9%); m.p. 210-211°C; IR (KBr) v 3381, 3310, 1694, 1621, 1470, 1352, 1251, 1225, 752, 704 cm^{-1}; ^1H NMR (400 MHz, $CDCl_3$) δ 8.63-8.72 (m, 1H), 7.49 (d, J = 6.8 Hz, 1H), 7.07-7.41 (m, 10H), 6.98 (d, J = 7.6 Hz, 1H), 6.86-6.92 (m, 2H), 5.20 (d, J = 7.2 Hz, 1H), 4.19 (t, J = 10.8 Hz, 1H), 4.03 (dd, J = 10.8, 2.4 Hz, 1H), 2.53 (s, 1H), 1.61 (s, 3H); ^{13}C NMR (100 MHz, DMSO-d_6) δ 205.3, 182.1, 156.0, 142.7, 142.3, 131.4, 129.9, 129.3, 128.3, 127.8, 127.5, 127.4, 126.1, 125.5, 122.2, 119.5, 115.8, 109.9, 68.0, 66.5, 66.2, 49.4, 30.6; HRMS (TOF-ES+) m/z: $[M+Na]^+$ calcd for $C_{25}H_{22}N_2O_3Na$ 421.1528, found 421.1518.

4'-Acetyl-3'-(2-hydroxyphenyl)-5'-phenylspiro[indoline-3,2'-pyrrolidin]-2-one (5f)

White solid (conditions A: 25.7 mg, yield 13%; conditions B: 141.7 mg, yield 71%); m.p. 213-215°C; IR (KBr) v 3376, 3256, 3189, 3078, 1702, 1673, 1471, 1455, 1361, 1257, 753, 704 cm^{-1}; ^1H NMR (400 MHz, DMSO-d_6) δ 9.91 (s, 1H), 8.96 (s, 1H), 7.62 (d, J = 7.2 Hz, 1H), 7.58 (d, J = 7.2 Hz, 2H), 7.29-7.36 (m, 3H), 7.25 (t, J = 7.2 Hz, 1H), 7.09 (td, J = 7.6, 1.2 Hz, 1H), 6.99 (t, J = 7.6 Hz, 1H), 6.87 (td, J = 8.0, 1.6 Hz, 1H), 6.62 (t, J = 7.2 Hz, 1H), 6.51-6.55 (m, 2H), 5.45 (dd, J = 10.4, 5.6 Hz, 1H), 4.83 (d, J = 12.0 Hz, 1H), 4.62 (t, J = 10.8 Hz, 1H), 3.98 (d, J = 6.0 Hz, 1H), 1.53 (s,

3H); ^{13}C NMR (100MHz, DMSO-d_6) δ 207.2, 181.5, 156.3, 143.6, 142.6, 130.9, 128.7, 128.6, 128.2, 127.9, 127.6, 125.5, 122.6, 121.6, 118.9, 115.2, 109.1, 72.1, 60.5, 60.3, 45.1, 30.9; HRMS (TOF-ES+) m/z: $[M+H]^+$ calcd for $C_{25}H_{23}N_2O_3$ 399.1709, found 399.1700.

3'-Acetyl-5'-phenyl-4'-(pyridin-2-yl)spiro[indoline-3,2'-pyrrolidin]-2-one (**4g**)
White powder, combined yield of **4g** and **5g** (conditions A: 172.3 mg, yield 90%; conditions B: 160.9 mg, yield 84%); IR (KBr) *ν* 3334, 3083, 3060, 1712, 1619, 1471, 1187, 753, 701 cm^{-1}; ^{1}H NMR (400 MHz, DMSO-d_6) δ 9.12 (s, 1H), 8.58 (s, 1H), 7.07-7.55 (m, 12H), 6.96 (d, J = 7.6 Hz, 1H) 6.90 (d, J = 7.6 Hz, 1H), 5.03 (d, J = 10.0 Hz, 1H), 4.58 (d, J = 11.2 Hz, 1H), 4.08 (dd, J = 10.8, 8.0 Hz, 1H), 1.61 (s, 3H); HRMS (TOF-ES+) m/z: $[M+Na]^+$ calcd for $C_{24}H_{21}N_3O_2Na$ 406.1531, found 406.1538.

*Note: Compounds **4g** and **5g** cannot be separated by column chromatography. Pure **4g** was obtained by recrystalization and the regioisomeric ratio was determined by ^{1}H NMR spectroscopy of the mixture of **4g** and **5g**.*

4'-Acetyl-5'-phenyl-3'-(pyridin-2-yl)spiro[indoline-3,2'-pyrrolidin]-2-one (**5g**)
White crystals; m.p. 209-211°C; IR (KBr) *ν* 3420, 3339, 3191, 3084, 1707, 1618, 1472, 1190, 754 cm^{-1}; ^{1}H NMR (400 MHz, DMSO-d_6) δ 9.91 (s, 1H), 8.35 (d, J = 4.4 Hz, 1H), 7.69 (d, J = 7.2 Hz, 1H), 7.08-7.58 (m, 9H), 6.65 (d, J = 7.6 Hz, 1H), 6.49 (d, J = 8.0 Hz, 1H), 5.43 (dd, J = 10.4, 5.6 Hz, 1H), 5.09 (t, J = 11.2 Hz, 1H), 4.32 (d, J = 11.2 Hz, 1H), 4.08 (d, J = 5.6 Hz, 1H), 1.71 (s, 3H); ^{13}C NMR (100 MHz, DMSO-d_6) δ 206.9, 180.2, 156.2, 149.0, 143.7, 142.9, 136.2, 131.2, 129.3, 128.9, 128.2, 127.6,

124.3, 122.58, 122.50, 122.2, 109.6, 71.0, 59.8, 57.3, 55.5, 31.3; HRMS (TOF-ES+) m/z: $[M+Na]^+$ calcd for $C_{24}H_{21}N_3O_2Na$ 406.1531, found 406.1516.

3'-Acetyl-4'-(2-nitrophenyl)-5'-phenylspiro[indoline-3,2'-pyrrolidin]-2-one (**4h**)
White solid (conditions A: 131.6 mg, yield 62%; conditions B: 99.8 mg, yield 47%); m.p. 213-214°C; IR (KBr) *v* 3393, 3321, 1715, 1620, 1484, 1440, 1347, 1185, 844, 750, 702 cm^{-1}; ^1H NMR (400 MHz, DMSO-d_6) δ 10.65 (s, 1H), 8.13 (d, J = 8.4 Hz, 2H), 7.51 (d, J = 8.4 Hz, 2H), 7.20-7.30 (m, 7H), 7.02 (t, J = 7.6 Hz, 1H), 6.89 (d, J = 7.6 Hz, 1H), 4.88 (t, J = 4.4 Hz, 1H), 4.06 (d, J = 4.8 Hz, 1H), 3.91-3.92 (m, 2H), 1.47 (s, 3H); ^{13}C NMR (100 MHz, DMSO-d_6) δ 204.5, 182.2, 148.1, 146.9, 142.4, 141.3, 130.9, 130.1, 129.7, 128.6, 128.0, 127.5, 126.1, 124.0, 122.4, 110.1, 67.7, 67.33, 67.26, 54.9, 30.4; HRMS (TOF-ES+) m/z: $[M+H]^+$ calcd for $C_{25}H_{22}N_3O_4$ 428.1610, found 428.1606.

4'-Acetyl-3'-(2-nitrophenyl)-5'-phenylspiro[indoline-3,2'-pyrrolidin]-2-one (**5h**)
White solid (conditions A: 64.8 mg, yield 30%; conditions B: 81.7 mg, yield 38%); m.p. 213-214°C; IR (KBr) *v* 3339, 3155, 3074, 3033, 2891, 1701, 1622, 1521, 1471, 1347, 753, 703 cm^{-1}; ^1H NMR (400 MHz, DMSO-d_6) δ 9.97 (s, 1H), 7.98 (d, J = 8.4 Hz, 2H), 7.74 (d, J = 7.2 Hz, 1H), 7.60 (d, J = 7.2 Hz, 2H), 7.09-7.37 (m, 7H), 6.59 (d, J = 7.6 Hz, 1H), 5.52 (dd, J = 10.4, 5.6 Hz, 1H), 4.90 (t, J = 11.2 Hz, 1H), 4.31 (d, J = 11.6 Hz, 1H), 4.21 (d, J = 5.6 Hz, 1H), 1.68 (s, 3H); ^{13}C NMR (100 MHz, DMSO-d_6) δ 206.4, 180.4, 147.1, 144.5, 143.4, 142.5, 130.2, 129.7, 129.5, 129.0, 128.3, 127.9, 124.6, 123.4, 122.5, 109.7, 72.3, 60.2, 58.6, 53.8, 31.4; HRMS (TOF-ES+) m/z:

$[M+H]^+$ calcd for $C_{25}H_{22}N_3O_4$ 428.1610, found 428.1599.

3'-Acetyl-4'-(4-nitrophenyl)-5'-phenylspiro[indoline-3,2'-pyrrolidin]-2-one (**4i**)

White solid (conditions A: 139.0 mg, yield 65%; conditions B: 104.1 mg, yield 49%); m.p. 267-268°C; IR (KBr) *v* 3393, 3321, 2940, 2882, 1715, 1513, 1470, 1346, 1186, 750, 702 cm^{-1}; ^1H NMR (400 MHz, $CDCl_3$) δ 8.13 (d, J = 8.8 Hz, 2H), 7.70 (*br* s, 1H), 7.51 (d, J = 8.4 Hz, 2H), 7.11-7.34 (m, 8H), 7.13 (t, J = 7.6 Hz, 1H), 6.93 (d, J = 7.6 Hz, 1H), 5.05 (d, J = 10.4 Hz, 1H), 4.12 (t, J = 10.0 Hz, 1H), 3.96 (d, J = 10.8 Hz, 1H), 1.56 (s, 3H); ^{13}C NMR (100 MHz, $CDCl_3$) δ 205.8, 180.3, 147.4, 143.0, 141.1, 140.5, 129.8, 129.0, 128.8, 128.6, 128.3, 124.1, 123.4, 123.3, 110.0, 72.5, 61.0, 58.9, 54.6, 31.3; HRMS (TOF-ES+) m/z: $[M+Na]^+$ calcd for $C_{25}H_{21}N_3O_4Na$ 450.1430, found 450.1448.

4'-Acetyl-3'-(4-nitrophenyl)-5'-phenylspiro[indoline-3,2'-pyrrolidin]-2-one (**5i**)

White solid (conditions A: 59.6 mg, yield 28%; conditions B: 75.3 mg, yield 35%); m.p. 217-218°C; IR (KBr) *v* 3411, 3340, 3074, 2891, 1698, 1621, 1521, 1471, 1346, 849, 752, 702 cm^{-1}; ^1H NMR (400 MHz, $CDCl_3$) δ 7.95 (d, J = 8.8 Hz, 2H), 7.71 (d, J = 6.8 Hz, 1H), 7.53 (d, J = 7.6 Hz, 2H), 7.38 (t, J = 7.2 Hz, 2H), 7.18-7.33 (m, 6H), 6.65 (d, J = 7.6 Hz, 1H), 5.68 (d, J = 10.8 Hz, 1H), 4.75 (t, J = 11.8 Hz, 1H), 4.44 (d, J = 11.2 Hz, 1H), 1.68 (s, 3H); ^{13}C NMR (100 MHz, DMSO-d_6) δ 206.3, 180.3, 147.1, 144.5, 143.3, 142.5, 130.2, 129.7, 129.5, 128.9, 128.3, 127.9, 124.5, 123.3, 122.4, 109.8, 72.4, 60.3, 58.7, 53.9, 31.4; HRMS (TOF-ES+) m/z: $[M+H]^+$ calcd for $C_{25}H_{22}N_3O_4$ 428.1610, found 428.1626.

3'-Acetyl-5'-phenyl-4'-(*o*-tolyl)spiro[indoline-3,2'-pyrrolidin]-2-one (4j)

White solid (conditions A: 142.1 mg, yield 72%; conditions B: 110.5 mg, yield 56%); m.p. 200-202°C; IR (KBr) v 3346, 3195, 1710, 1623, 1471, 1357, 1180, 743, 702 cm^{-1}; ^{1}H NMR (400 MHz, $CDCl_3$) δ 9.20-9.28 (m, 1H), 7.67 (d, J = 8.0 Hz, 1H), 7.39 (d, J = 7.6 Hz, 1H), 7.19-7.31 (m, 7H), 7.13 (t, J = 7.6 Hz, 1H), 7.07 (t, J = 7.6 Hz, 1H), 6.99 (t, J = 8.0 Hz, 2H), 4.90 (d, J = 8.0 Hz, 1H), 4.27 (t, J = 10.8 Hz, 1H), 4.08 (d, J = 10.4 Hz, 1H), 2.60 (s, 1H), 1.98 (s, 3H), 1.55 (s, 3H); ^{13}C NMR (100 MHz, $CDCl_3$) δ 204.7, 183.5, 140.54, 140.48, 140.45, 137.9, 137.2, 130.3, 130.2, 129.7, 128.3, 127.7, 126.7, 126.6, 126.3, 126.1, 123.6, 110.4, 69.5, 68.2, 68.0, 50.3, 30.7, 19.6; HRMS (TOF-ES+) m/z: $[M+Na]^+$ calcd for $C_{26}H_{24}N_2O_2Na$ 419.1735, found 419.1739.

4'-Acetyl-5'-phenyl-3'-(*o*-tolyl)spiro[indoline-3,2'-pyrrolidin]-2-one (5j)

White solid (conditions A: 40.1 mg, yield 20%; conditions B: 73.7 mg, yield 37%); m.p. 206-207°C; IR (KBr) v 3380, 3203, 1709, 1620, 1471, 1360, 750, 700 cm^{-1}; ^{1}H NMR (400 MHz, DMSO-d_6) δ 9.98 (s, 1H), 7.64 (d, J = 7.2 Hz, 1H), 7.59 (d, J = 6.8 Hz, 2H), 7.51 (d, J = 7.6 Hz, 1H), 7.35 (t, J = 7.2 Hz, 2H), 7.26 (t, J = 7.2 Hz, 1H), 6.96-7.14 (m, 4H), 6.89 (d, J = 7.2 Hz, 1H), 6.55 (d, J = 7.2 Hz, 1H), 5.57 (dd, J = 8.8, 6.0 Hz, 1H), 4.55-4.63 (m, 2H), 4.08 (d, J = 5.6 Hz, 1H), 2.03 (s, 3H), 1.55 (s, 3H); ^{13}C NMR (100 MHz, DMSO-d_6) δ 206.9, 181.7, 143.5, 142.6, 137.7, 135.2, 130.8, 130.4, 129.2, 128.9, 128.3, 127.8, 127.4, 127.0, 126.1, 124.8, 121.6, 109.6, 72.4, 61.8, 60.5, 48.9, 31.6, 20.1; HRMS (TOF-ES+) m/z: $[M+Na]^+$ calcd for $C_{26}H_{24}N_2O_2Na$ 419.1735, found 419.1714.

3'-Acetyl-4'-(4-hydroxyphenyl)-5'-phenylspiro[indoline-3,2'-pyrrolidin]-2-one (4k)

White solid (conditions A: 187.1 mg, yield 94%; conditions B: 162.4 mg, yield 82%); m.p. 230-231°C; IR (KBr) ν 3332, 3060, 3030, 1717, 1619, 1470, 1187, 752, 699 cm^{-1}; ^1H NMR (400 MHz, DMSO-d_6) δ 10.64 (s, 1H), 9.30 (s, 1H), 7.39-7.41 (m, 7H), 7.05-7.21 (m, 3H), 6.95 (d, J = 7.6 Hz, 1H), 6.73 (d, J = 8.4 Hz, 2H), 4.84 (dd, J = 10.0, 5.2 Hz, 1H), 3.93 (d, J = 5.2 Hz, 1H), 3.83 (d, J = 10.8 Hz, 1H), 3.74 (t, J = 10.4 Hz, 1H), 1.56 (s, 3H). ^{13}C NMR (100 MHz, DMSO-d_6) δ 204.9, 182.5, 156.5, 142.3, 131.3, 129.8, 129.5, 129.4, 128.4, 127.6, 127.5, 126.2, 122.3, 115.6, 110.0, 67.8, 67.7, 67.3, 54.3, 30.8; HRMS (TOF-ES+) m/z: $[M+Na]^+$ calcd for $C_{25}H_{22}N_2O_3Na$ 421.1528, found 421.1547.

References

(1) Titu, D.; Chadha, A. *Tetrahedron: Asymmetry*, **2008**, *19*, 1698-1701.

Copies of NMR spectra for spiropyrrolidine oxindoles

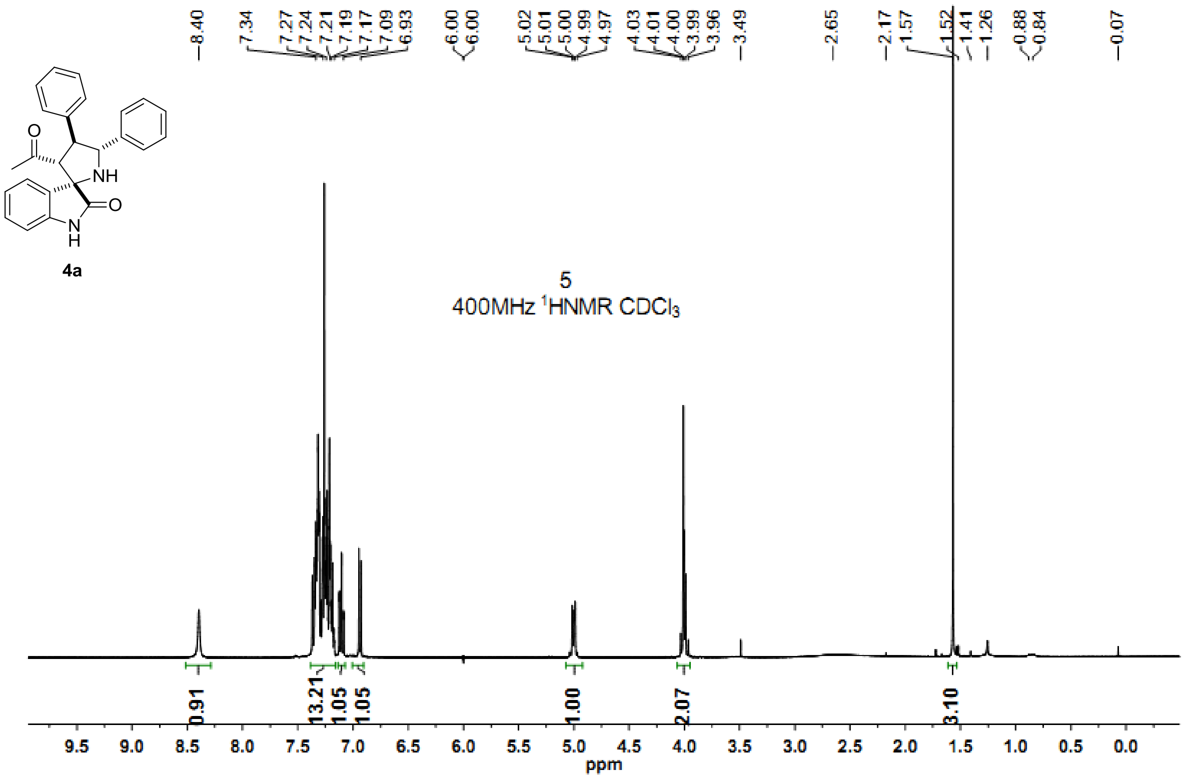

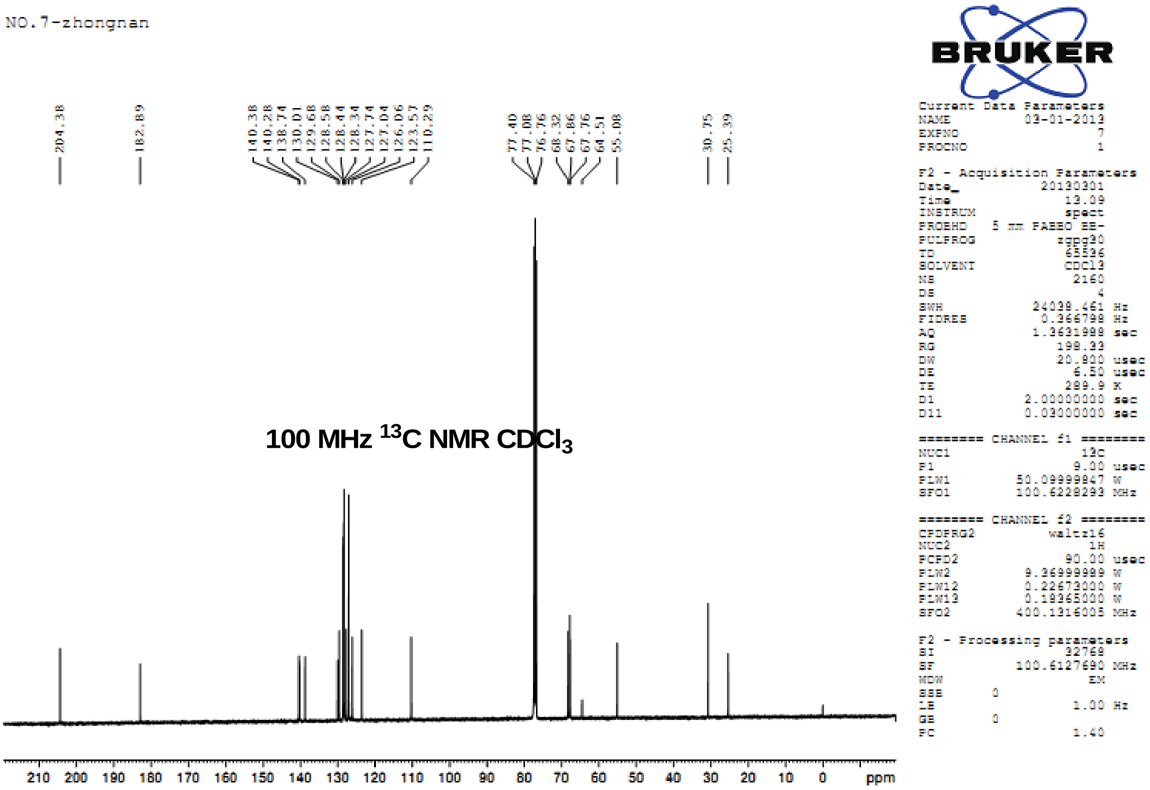

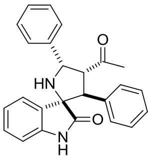

5a

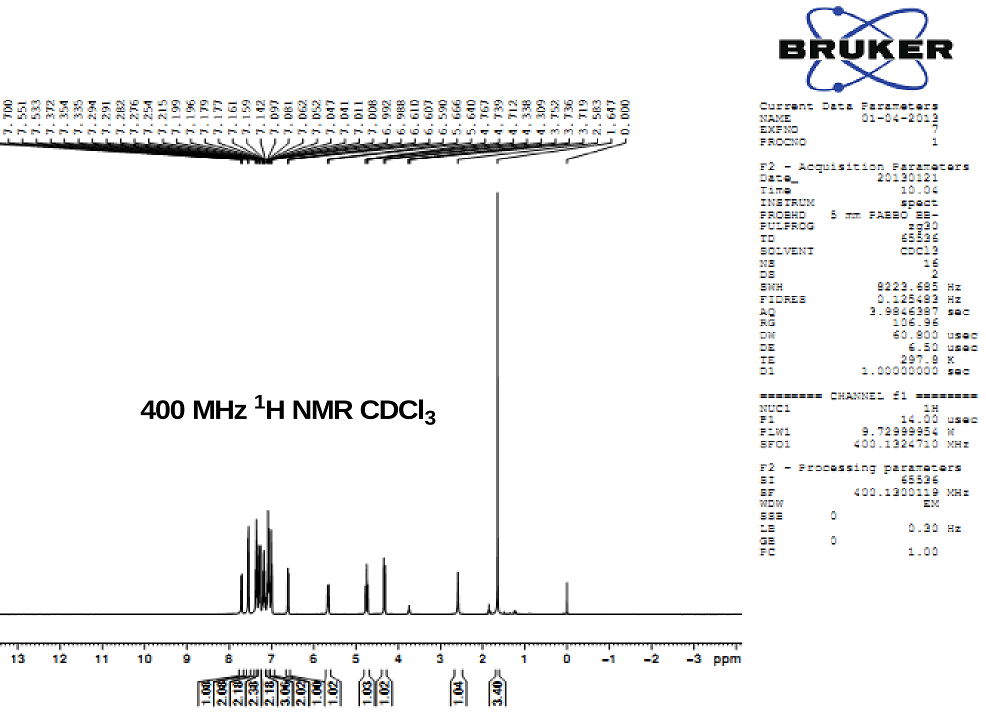
400 MHz ^{1}H NMR $CDCl_3$
7.721 7.704 7.700 7.551 7.533 7.372 7.354 7.335 7.294 7.291 7.282 7.276 7.254 7.215 7.199 7.196 7.179 7.177 7.161 7.159 7.142 7.097 7.081 7.062 7.052 7.047 7.041 7.011 7.008 6.992 6.988 6.610 6.607 6.590 5.666 5.640 4.767 4.739 4.712 4.338 4.309 3.752 3.736 3.719 2.583 1.647 0.000
16 15 14 13 12 11 10 9 8 7 6 5 4 3 2 1 0 -1 -2 -3 ppm
1.00 2.08 2.10 2.38 2.18 3.06 2.02 1.00 1.02 1.03 1.02 1.04 3.40
BRUKER
Current Data Parameters
NAME 01-04-2013
EXPNO 7
PROCNO 1
F2 - Acquisition Parameters
Date_ 20130121
Time 10.04
INSTRUM spect
PROBHD 5 mm PABBO BB-
PULPROG zg30
TD 65536
SOLVENT CDCl3
NS 16
DS 2
SWH 8223.685 Hz
FIDRES 0.125483 Hz
AQ 3.9846387 sec
RG 106.96
DW 60.800 usec
DE 6.50 usec
TE 297.9 K
D1 1.00000000 sec
======== CHANNEL f1 ========
NUC1 1H
P1 14.00 usec
PLW1 9.72999954 W
SFO1 400.1324710 MHz
F2 - Processing parameters
SI 65536
SF 400.1300119 MHz
WDW EM
SSB 0
LB 0.30 Hz
GB 0
PC 1.00

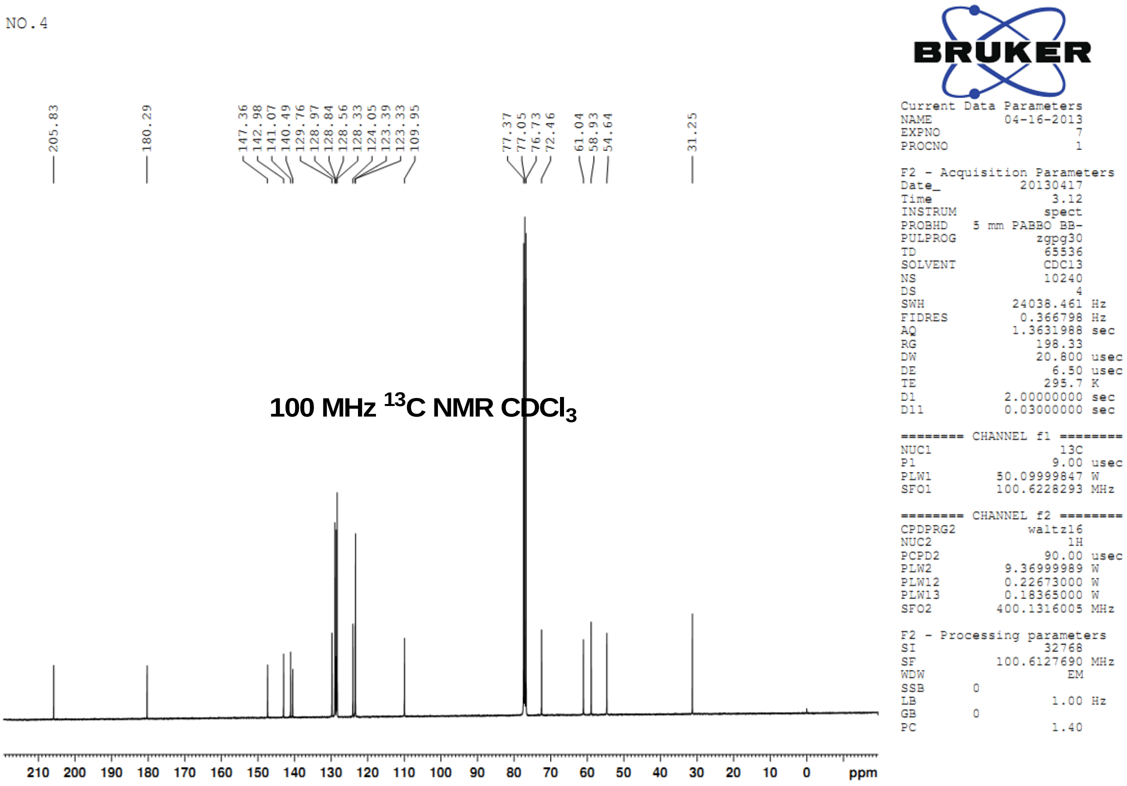
NO.4
205.83
180.29
147.36 142.98 141.07 140.49 129.76 128.97 128.84 128.56 128.33 124.05 123.39 123.33 109.95
77.37 77.05 76.73 72.46
61.04 58.93 54.64
31.25
100 MHz ^{13}C NMR $CDCl_3$
210 200 190 180 170 160 150 140 130 120 110 100 90 80 70 60 50 40 30 20 10 0 ppm
BRUKER
Current Data Parameters
NAME 04-16-2013
EXPNO 7
PROCNO 1
F2 - Acquisition Parameters
Date_ 20130417
Time 3.12
INSTRUM spect
PROBHD 5 mm PABBO BB-
PULPROG zgpg30
TD 65536
SOLVENT CDCl3
NS 10240
DS 4
SWH 24038.461 Hz
FIDRES 0.366798 Hz
AQ 1.3631988 sec
RG 198.33
DW 20.800 usec
DE 6.50 usec
TE 295.7 K
D1 2.00000000 sec
D11 0.03000000 sec
======== CHANNEL f1 ========
NUC1 13C
P1 9.00 usec
PLW1 50.09999847 W
SFO1 100.6228293 MHz
======== CHANNEL f2 ========
CPDPRG2 waltz16
NUC2 1H
PCPD2 90.00 usec
PLW2 9.36999989 W
PLW12 0.22673000 W
PLW13 0.16365000 W
SFO2 400.1316005 MHz
F2 - Processing parameters
SI 32768
SF 100.6127690 MHz
WDW EM
SSB 0
LB 1.00 Hz
GB 0
PC 1.40

PC-10

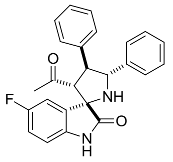

4b

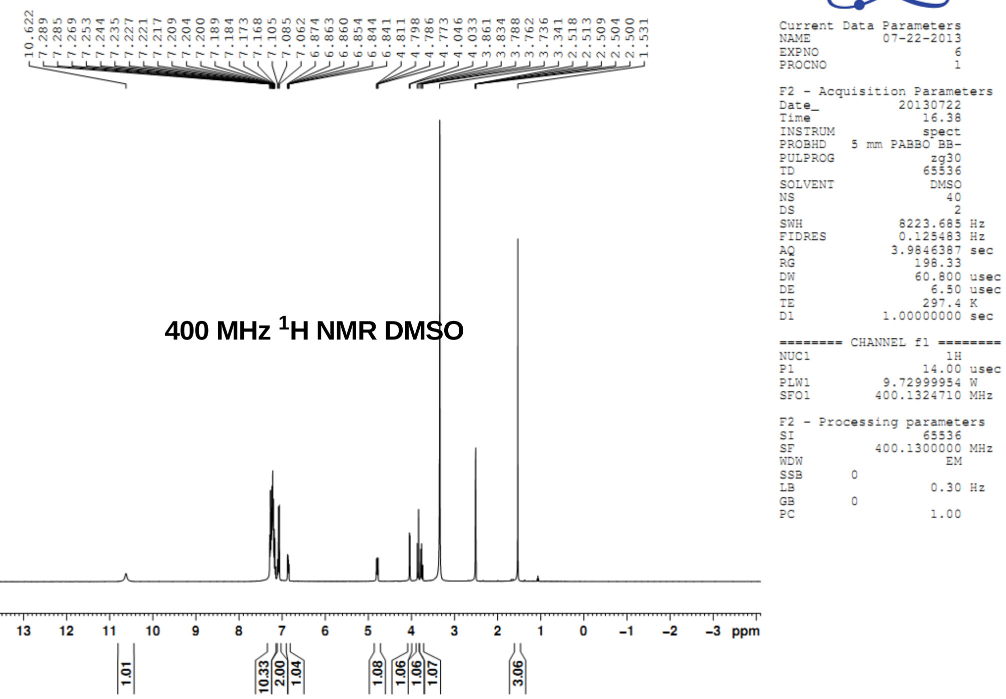
400 MHz ¹H NMR DMSO
10.622 7.289 7.285 7.269 7.253 7.244 7.235 7.227 7.221 7.217 7.209 7.204 7.200 7.189 7.184 7.173 7.168 7.105 7.085 7.062 6.874 6.863 6.860 6.854 6.844 6.841 4.811 4.798 4.786 4.773 4.046 4.033 3.861 3.834 3.788 3.762 3.736 3.341 2.518 2.513 2.509 2.504 2.500 1.531
16 15 14 13 12 11 10 9 8 7 6 5 4 3 2 1 0 −1 −2 −3 ppm
1.01 10.33 2.00 1.04 1.08 1.06 1.06 1.07 3.06
Current Data Parameters
NAME 07-22-2013
EXPNO 6
PROCNO 1
F2 - Acquisition Parameters
Date_ 20130722
Time 16.38
INSTRUM spect
PROBHD 5 mm PABBO BB-
PULPROG zg30
TD 65536
SOLVENT DMSO
NS 40
DS 2
SWH 8223.685 Hz
FIDRES 0.125483 Hz
AQ 3.9846387 sec
RG 198.33
DW 60.800 usec
DE 6.50 usec
TE 297.4 K
D1 1.00000000 sec
======== CHANNEL f1 ========
NUC1 1H
P1 14.00 usec
PLW1 9.72999954 W
SFO1 400.1324710 MHz
F2 - Processing parameters
SI 65536
SF 400.1300000 MHz
WDW EM
SSB 0
LB 0.30 Hz
GB 0
PC 1.00

PC-10

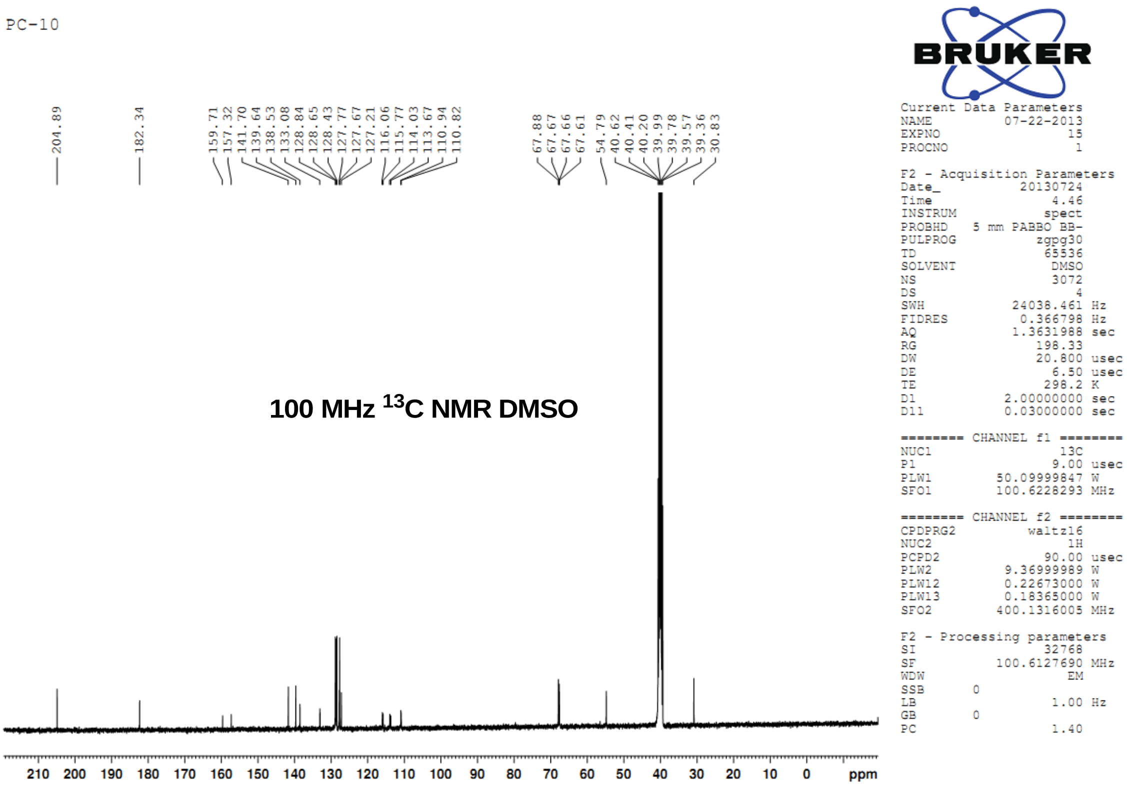
100 MHz ¹³C NMR DMSO
204.89 182.34 159.71 157.32 141.70 139.64 138.53 133.08 128.84 128.65 128.43 127.77 127.67 127.21 116.06 115.77 114.03 113.67 110.94 110.82 67.88 67.67 67.66 67.61 54.79 40.62 40.41 40.20 39.99 39.78 39.57 39.36 30.83
210 200 190 180 170 160 150 140 130 120 110 100 90 80 70 60 50 40 30 20 10 0 ppm
Current Data Parameters
NAME 07-22-2013
EXPNO 15
PROCNO 1
F2 - Acquisition Parameters
Date_ 20130724
Time 4.46
INSTRUM spect
PROBHD 5 mm PABBO BB-
PULPROG zgpg30
TD 65536
SOLVENT DMSO
NS 3072
DS 4
SWH 24038.461 Hz
FIDRES 0.366798 Hz
AQ 1.3631988 sec
RG 198.33
DW 20.800 usec
DE 6.50 usec
TE 298.2 K
D1 2.00000000 sec
D11 0.03000000 sec
======== CHANNEL f1 ========
NUC1 13C
P1 9.00 usec
PLW1 50.09999847 W
SFO1 100.6228293 MHz
======== CHANNEL f2 ========
CPDPRG2 waltz16
NUC2 1H
PCPD2 90.00 usec
PLW2 9.36999989 W
PLW12 0.22673000 W
PLW13 0.18365000 W
SFO2 400.1316005 MHz
F2 - Processing parameters
SI 32768
SF 100.6127690 MHz
WDW EM
SSB 0
LB 1.00 Hz
GB 0
PC 1.40

PC-7

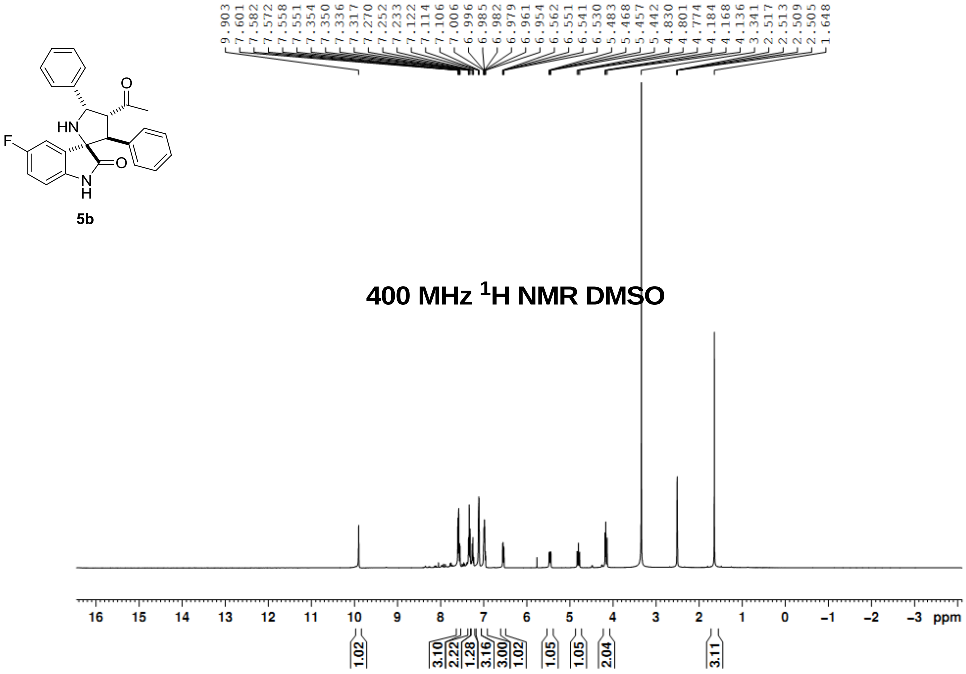


```
Current Data Parameters
NAME          07-22-2013
EXPNO                  3
PROCNO                 1

F2 - Acquisition Parameters
Date_           20130722
Time               15.28
INSTRUM            spect
PROBHD   5 mm PABBO BB-
PULPROG             zg30
TD                 65536
SOLVENT             DMSO
NS                    64
DS                     2
SWH             8223.685 Hz
FIDRES          0.125483 Hz
AQ             3.9846387 sec
RG                198.33
DW                60.800 usec
DE                  6.50 usec
TE                 297.5 K
D1            1.00000000 sec

======== CHANNEL f1 ========
NUC1                  1H
P1                 14.00 usec
PLW1          9.72999954 W
SFO1         400.1324710 MHz

F2 - Processing parameters
SI                 65536
SF           400.1300000 MHz
WDW                   EM
SSB      0
LB                  0.30 Hz
GB       0
PC                  1.00
```

PC-7

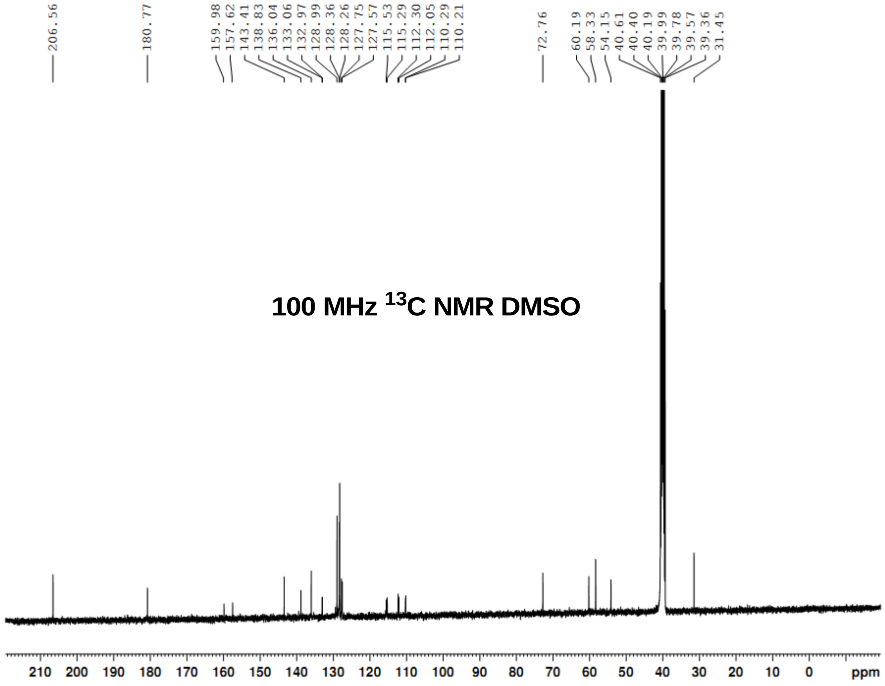


```
Current Data Parameters
NAME          07-22-2013
EXPNO                 13
PROCNO                 1

F2 - Acquisition Parameters
Date_           20130723
Time               19.11
INSTRUM            spect
PROBHD   5 mm PABBO BB-
PULPROG           zgpg30
TD                 65536
SOLVENT             DMSO
NS                  3000
DS                     4
SWH            24038.461 Hz
FIDRES          0.366798 Hz
AQ             1.3631988 sec
RG                168.03
DW                20.800 usec
DE                  6.50 usec
TE                 297.9 K
D1            2.00000000 sec
D11           0.03000000 sec

======== CHANNEL f1 ========
NUC1                 13C
P1                  9.00 usec
PLW1         50.09999847 W
SFO1         100.6228293 MHz

======== CHANNEL f2 ========
CPDPRG2           waltz16
NUC2                  1H
PCPD2              90.00 usec
PLW2          9.36999989 W
PLW12         0.22673000 W
PLW13         0.18365000 W
SFO2         400.1316005 MHz

F2 - Processing parameters
SI                 32768
SF           100.6127690 MHz
WDW                   EM
SSB      0
LB                  1.00 Hz
GB       0
PC                  1.40
```

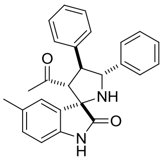

4c

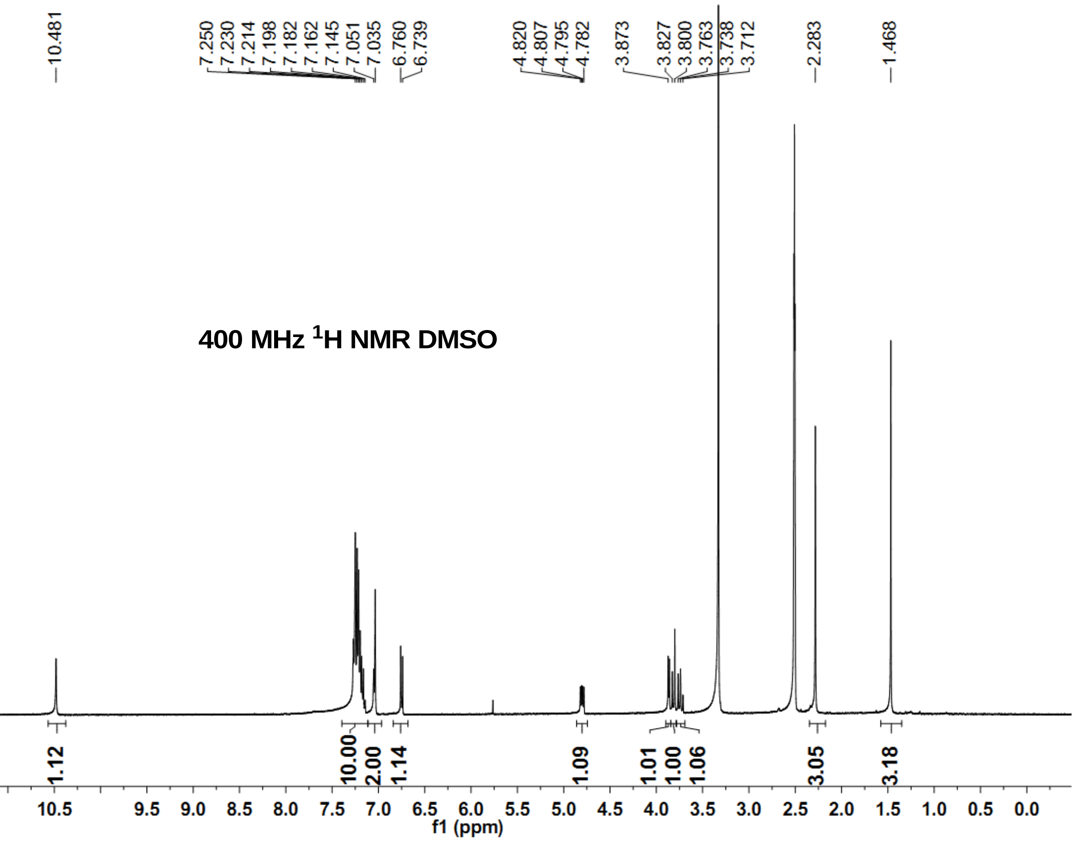

PC-16

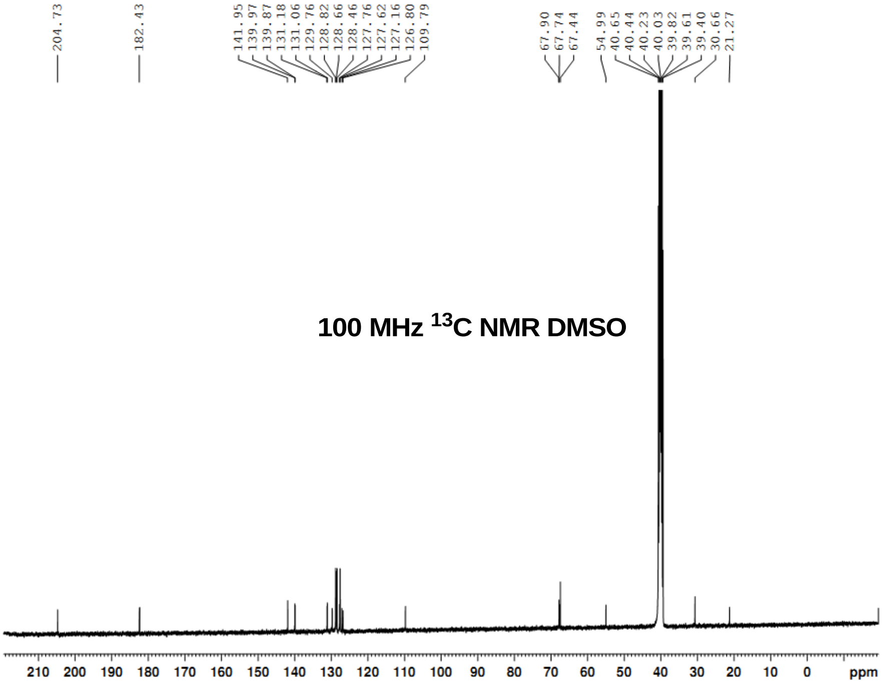


```
Current Data Parameters
NAME          08-12-2013
EXPNO                  6
PROCNO                 1

F2 - Acquisition Parameters
Date_           20130813
Time                9.18
INSTRUM            spect
PROBHD   5 mm PABBO BB-
PULPROG          zgpg30
TD                 65536
SOLVENT             DMSO
NS                 14352
DS                     4
SWH            24038.461 Hz
FIDRES          0.366798 Hz
AQ             1.3631988 sec
RG                152.51
DW                20.800 usec
DE                  6.50 usec
TE                 298.7 K
D1            2.00000000 sec
D11           0.03000000 sec

======== CHANNEL f1 ========
NUC1                 13C
P1                  9.00 usec
PLW1         50.09999847 W
SFO1        100.6228293 MHz

======== CHANNEL f2 ========
CPDPRG2          waltz16
NUC2                  1H
PCPD2              90.00 usec
PLW2          9.36999989 W
PLW12         0.22673000 W
PLW13         0.18365000 W
SFO2        400.1316005 MHz

F2 - Processing parameters
SI                 32768
SF           100.6127690 MHz
WDW                   EM
SSB      0
LB                  1.00 Hz
GB       0
PC                  1.40
```

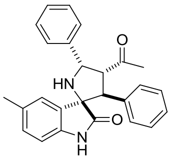
HN
O
N
H
O

5c

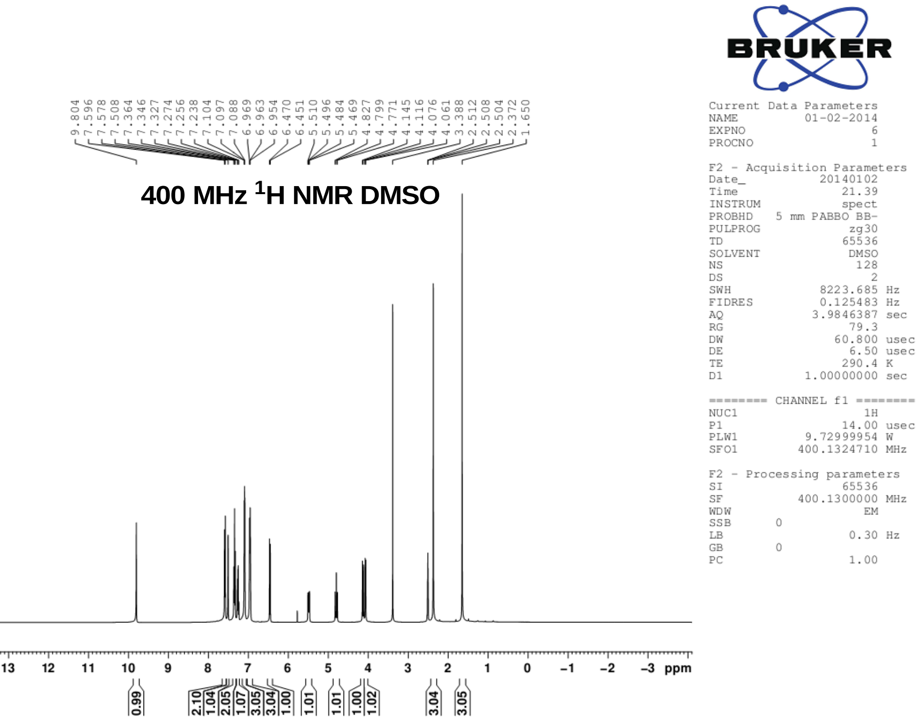
PC
9.804
7.596
7.578
7.508
7.364
7.346
7.327
7.274
7.256
7.238
7.104
7.097
7.088
6.969
6.963
6.954
6.470
6.451
5.510
5.496
5.484
5.469
4.827
4.799
4.771
4.145
4.116
4.076
4.061
3.388
2.512
2.508
2.504
2.372
1.650
400 MHz 1H NMR DMSO
16 15 14 13 12 11 10 9 8 7 6 5 4 3 2 1 0 −1 −2 −3 ppm
0.99
2.10
1.04
2.05
1.07
3.05
3.04
1.00
1.01
1.01
1.00
1.02
3.04
3.05
BRUKER
Current Data Parameters
NAME 01-02-2014
EXPNO 6
PROCNO 1
F2 - Acquisition Parameters
Date_ 20140102
Time 21.39
INSTRUM spect
PROBHD 5 mm PABBO BB-
PULPROG zg30
TD 65536
SOLVENT DMSO
NS 128
DS 2
SWH 8223.685 Hz
FIDRES 0.125483 Hz
AQ 3.9846387 sec
RG 79.3
DW 60.800 usec
DE 6.50 usec
TE 290.4 K
D1 1.00000000 sec
======== CHANNEL f1 ========
NUC1 1H
P1 14.00 usec
PLW1 9.72999954 W
SFO1 400.1324710 MHz
F2 - Processing parameters
SI 65536
SF 400.1300000 MHz
WDW EM
SSB 0
LB 0.30 Hz
GB 0
PC 1.00

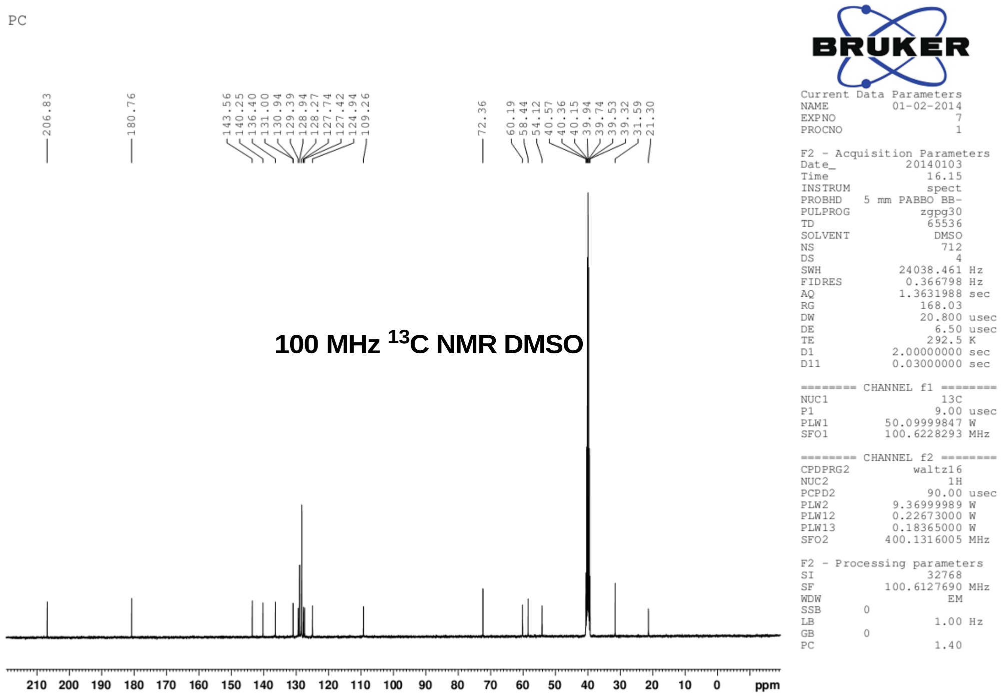
PC
206.83
180.76
143.56
140.25
136.40
131.00
130.94
129.39
128.94
128.27
127.74
127.42
124.94
109.26
72.36
60.19
58.44
54.12
40.57
40.36
40.15
39.94
39.74
39.53
39.32
31.59
21.30
100 MHz 13C NMR DMSO
210 200 190 180 170 160 150 140 130 120 110 100 90 80 70 60 50 40 30 20 10 0 ppm
BRUKER
Current Data Parameters
NAME 01-02-2014
EXPNO 7
PROCNO 1
F2 - Acquisition Parameters
Date_ 20140103
Time 16.15
INSTRUM spect
PROBHD 5 mm PABBO BB-
PULPROG zgpg30
TD 65536
SOLVENT DMSO
NS 712
DS 4
SWH 24038.461 Hz
FIDRES 0.366798 Hz
AQ 1.3631988 sec
RG 168.03
DW 20.800 usec
DE 6.50 usec
TE 292.5 K
D1 2.00000000 sec
D11 0.03000000 sec
======== CHANNEL f1 ========
NUC1 13C
P1 9.00 usec
PLW1 50.09999847 W
SFO1 100.6228293 MHz
======== CHANNEL f2 ========
CPDPRG2 waltz16
NUC2 1H
PCPD2 90.00 usec
PLW2 9.36999989 W
PLW12 0.22673000 W
PLW13 0.18365000 W
SFO2 400.1316005 MHz
F2 - Processing parameters
SI 32768
SF 100.6127690 MHz
WDW EM
SSB 0
LB 1.00 Hz
GB 0
PC 1.40

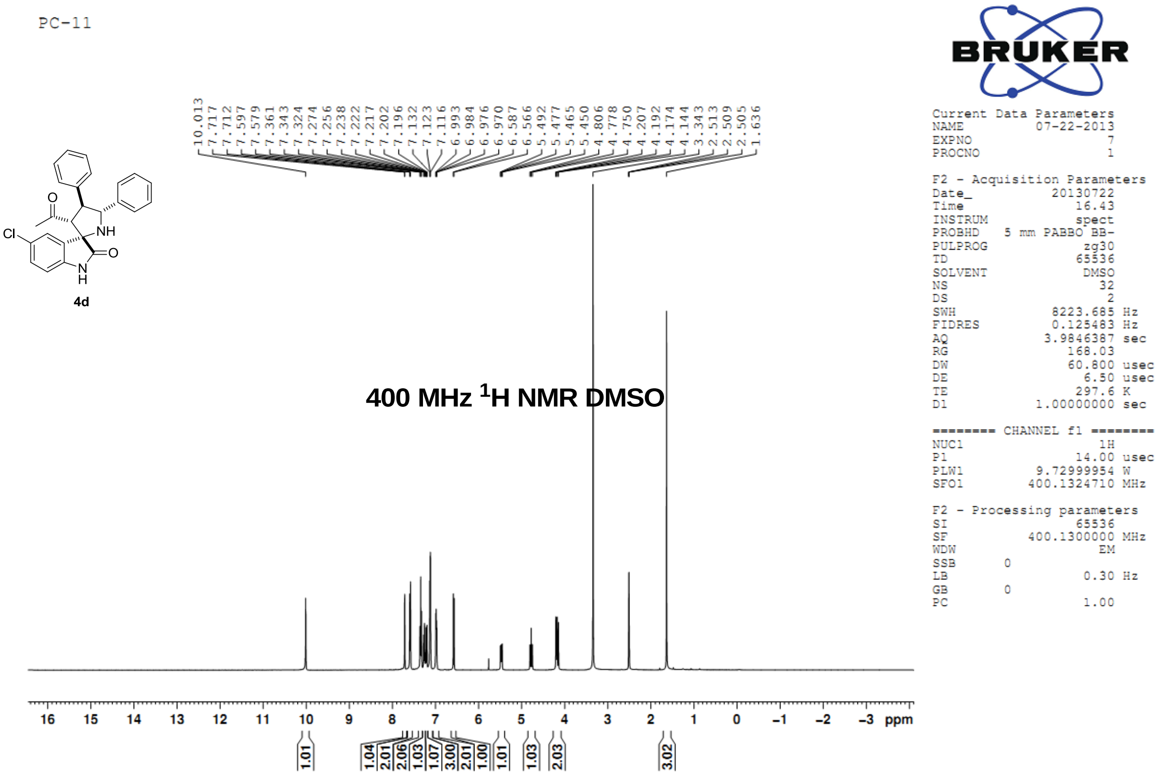
PC-11
4d
400 MHz ¹H NMR DMSO
10.013 7.717 7.712 7.597 7.579 7.361 7.343 7.324 7.274 7.256 7.238 7.222 7.217 7.202 7.196 7.132 7.123 7.116 6.993 6.984 6.976 6.970 6.587 6.566 5.492 5.477 5.465 5.450 4.806 4.778 4.750 4.207 4.192 4.174 4.144 3.343 2.513 2.509 2.505 1.636
1.01 1.04 2.01 2.06 1.03 1.07 3.00 2.01 1.00 1.01 1.03 2.03 3.02
16 15 14 13 12 11 10 9 8 7 6 5 4 3 2 1 0 −1 −2 −3 ppm
BRUKER
Current Data Parameters
NAME 07-22-2013
EXPNO 7
PROCNO 1
F2 - Acquisition Parameters
Date_ 20130722
Time 16.43
INSTRUM spect
PROBHD 5 mm PABBO BB-
PULPROG zg30
TD 65536
SOLVENT DMSO
NS 32
DS 2
SWH 8223.685 Hz
FIDRES 0.125483 Hz
AQ 3.9846387 sec
RG 168.03
DW 60.800 usec
DE 6.50 usec
TE 297.6 K
D1 1.00000000 sec
======== CHANNEL f1 ========
NUC1 1H
P1 14.00 usec
PLW1 9.72999954 W
SFO1 400.1324710 MHz
F2 - Processing parameters
SI 65536
SF 400.1300000 MHz
WDW EM
SSB 0
LB 0.30 Hz
GB 0
PC 1.00

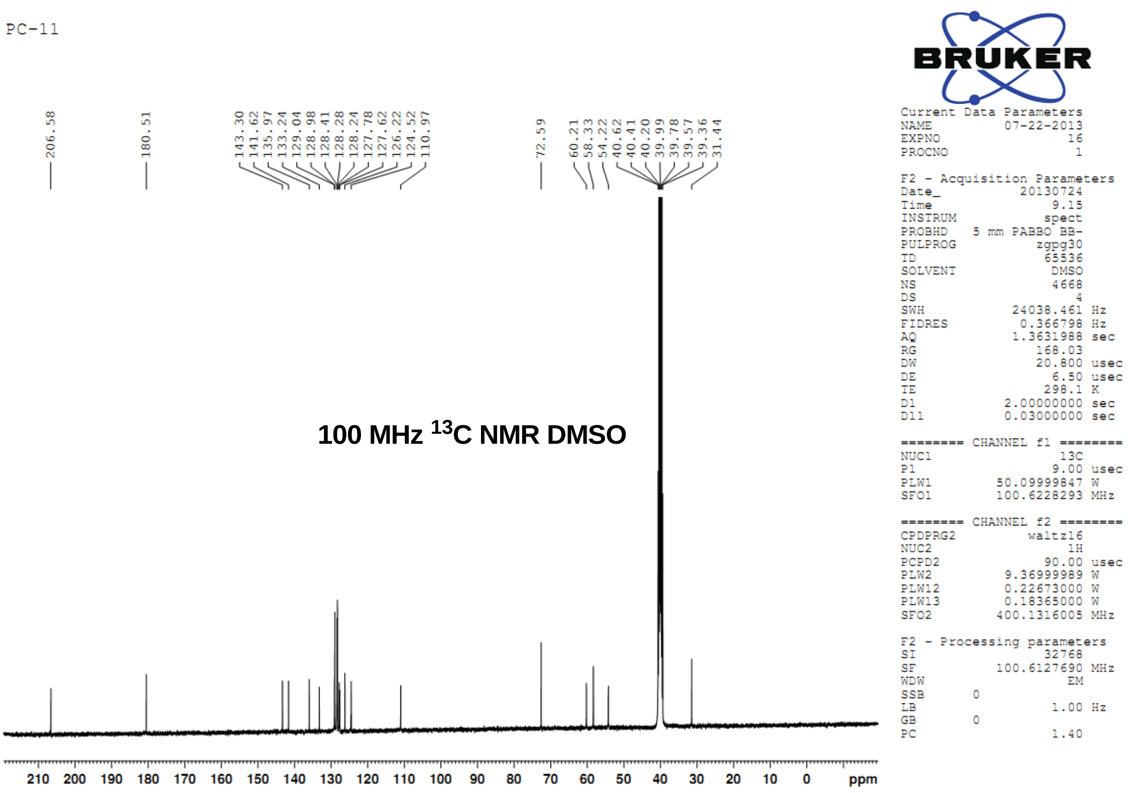
PC-11
100 MHz ¹³C NMR DMSO
206.58 180.51 143.30 141.62 135.97 133.24 129.04 128.98 128.41 128.28 128.24 127.78 127.62 126.22 124.52 110.97 72.59 60.21 58.33 54.22 40.62 40.41 40.20 39.99 39.78 39.57 39.36 31.44
210 200 190 180 170 160 150 140 130 120 110 100 90 80 70 60 50 40 30 20 10 0 ppm
BRUKER
Current Data Parameters
NAME 07-22-2013
EXPNO 16
PROCNO 1
F2 - Acquisition Parameters
Date_ 20130724
Time 9.15
INSTRUM spect
PROBHD 5 mm PABBO BB-
PULPROG zgpg30
TD 65536
SOLVENT DMSO
NS 4668
DS 4
SWH 24038.461 Hz
FIDRES 0.366798 Hz
AQ 1.3631988 sec
RG 168.03
DW 20.800 usec
DE 6.50 usec
TE 298.1 K
D1 2.00000000 sec
D11 0.03000000 sec
======== CHANNEL f1 ========
NUC1 13C
P1 9.00 usec
PLW1 50.09999847 W
SFO1 100.6228293 MHz
======== CHANNEL f2 ========
CPDPRG2 waltz16
NUC2 1H
PCPD2 90.00 usec
PLW2 9.36999989 W
PLW12 0.22673000 W
PLW13 0.18365000 W
SFO2 400.1316005 MHz
F2 - Processing parameters
SI 32768
SF 100.6127690 MHz
WDW EM
SSB 0
LB 1.00 Hz
GB 0
PC 1.40

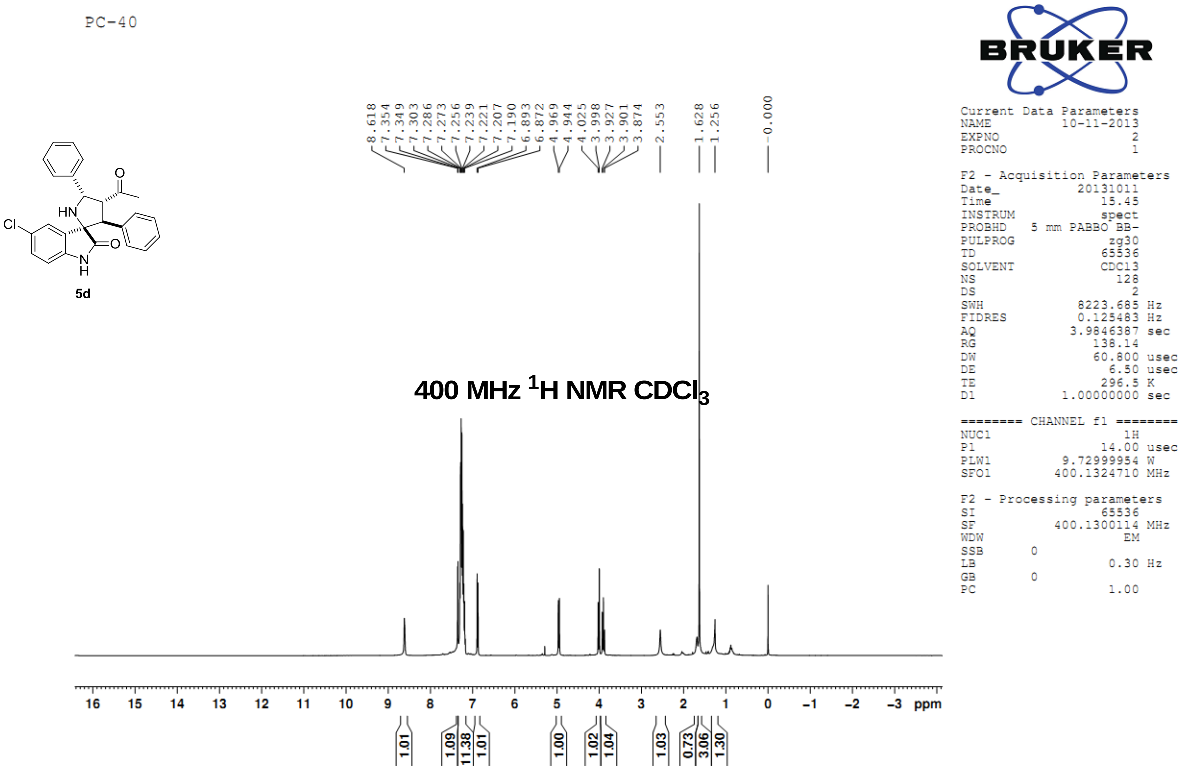
PC-40
5d
400 MHz 1H NMR CDCl3
ppm
Current Data Parameters
NAME 10-11-2013
EXPNO 2
PROCNO 1
F2 - Acquisition Parameters
Date_ 20131011
Time 15.45
INSTRUM spect
PROBHD 5 mm PABBO BB-
PULPROG zg30
TD 65536
SOLVENT CDCl3
NS 128
DS 2
SWH 8223.685 Hz
FIDRES 0.125483 Hz
AQ 3.9846387 sec
RG 138.14
DW 60.800 usec
DE 6.50 usec
TE 296.5 K
D1 1.00000000 sec
======== CHANNEL f1 ========
NUC1 1H
P1 14.00 usec
PLW1 9.72999954 W
SFO1 400.1324710 MHz
F2 - Processing parameters
SI 65536
SF 400.1300114 MHz
WDW EM
SSB 0
LB 0.30 Hz
GB 0
PC 1.00

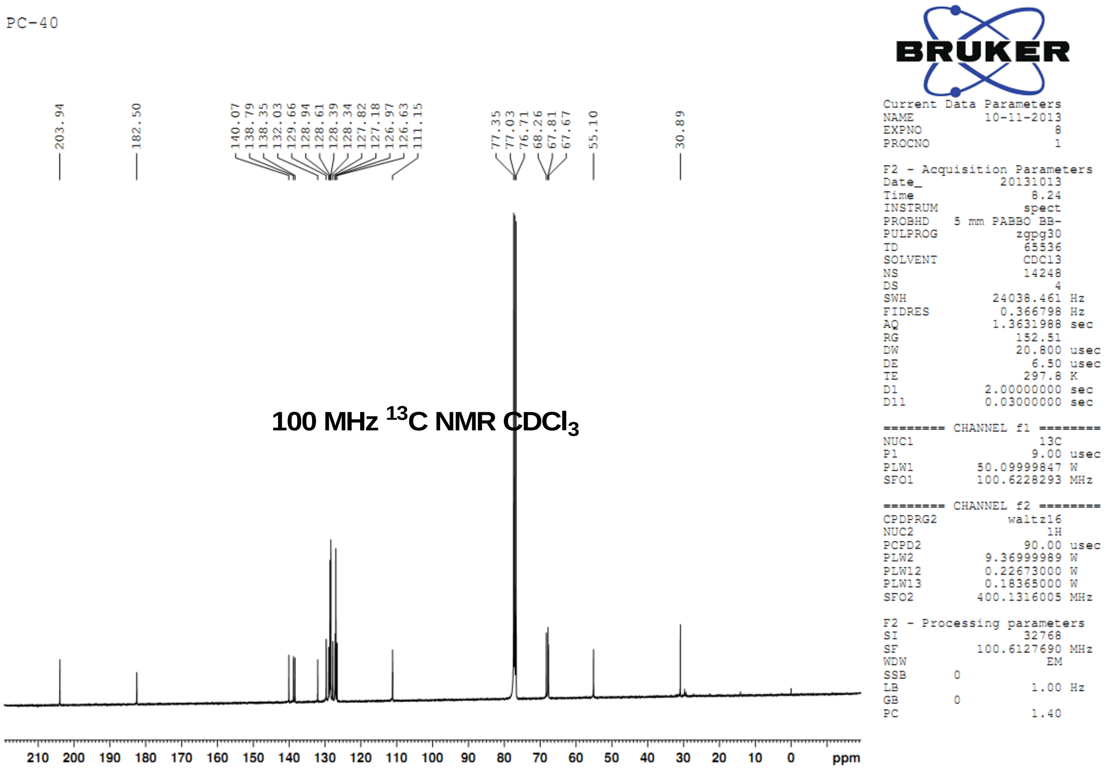
PC-40
100 MHz 13C NMR CDCl3
ppm
Current Data Parameters
NAME 10-11-2013
EXPNO 8
PROCNO 1
F2 - Acquisition Parameters
Date_ 20131013
Time 8.24
INSTRUM spect
PROBHD 5 mm PABBO BB-
PULPROG zgpg30
TD 65536
SOLVENT CDCl3
NS 14248
DS 4
SWH 24038.461 Hz
FIDRES 0.366798 Hz
AQ 1.3631988 sec
RG 152.51
DW 20.800 usec
DE 6.50 usec
TE 297.8 K
D1 2.00000000 sec
D11 0.03000000 sec
======== CHANNEL f1 ========
NUC1 13C
P1 9.00 usec
PLW1 50.09999847 W
SFO1 100.6228293 MHz
======== CHANNEL f2 ========
CPDPRG2 waltz16
NUC2 1H
PCPD2 90.00 usec
PLW2 9.36999989 W
PLW12 0.22673000 W
PLW13 0.18365000 W
SFO2 400.1316005 MHz
F2 - Processing parameters
SI 32768
SF 100.6127690 MHz
WDW EM
SSB 0
LB 1.00 Hz
GB 0
PC 1.40

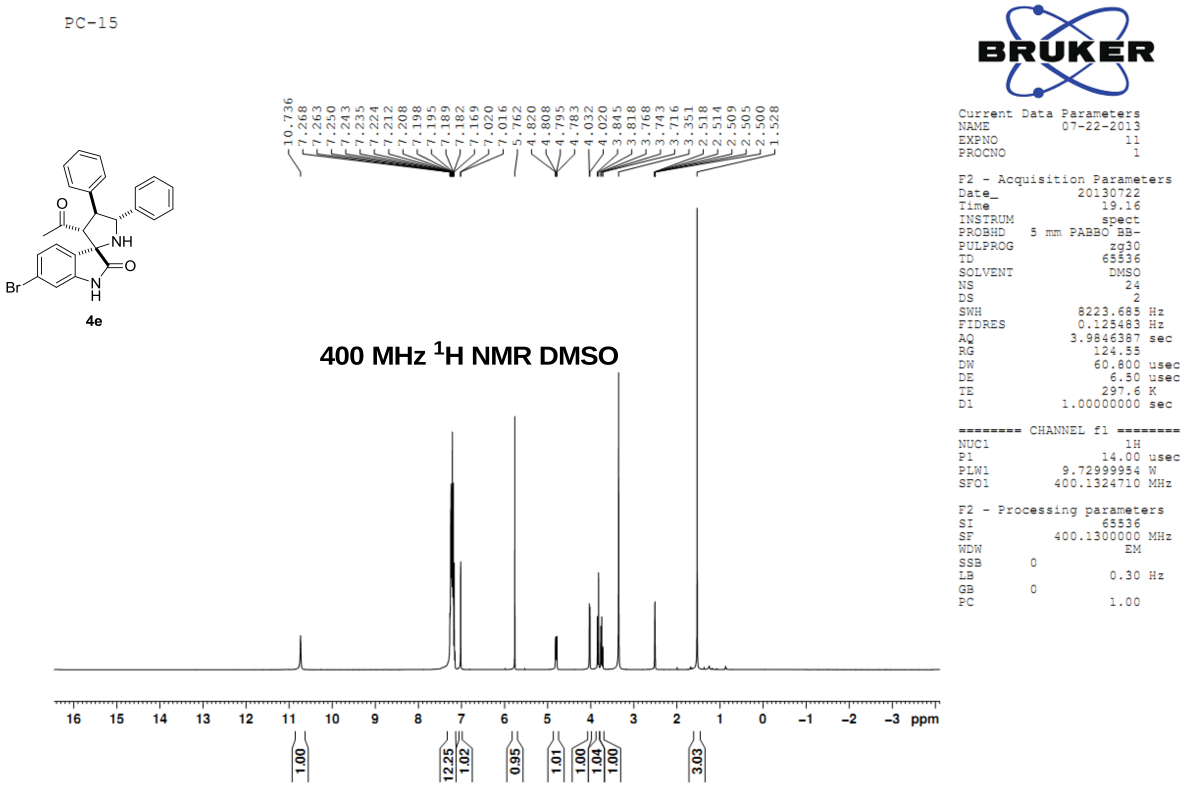

PC-15
Br
NH
N
H
O
O
4e
10.736
7.268
7.263
7.250
7.243
7.235
7.224
7.212
7.208
7.198
7.195
7.189
7.182
7.169
7.020
7.016
5.762
4.820
4.808
4.795
4.783
4.032
4.020
3.845
3.818
3.768
3.743
3.716
3.351
2.518
2.514
2.509
2.505
2.500
1.528
400 MHz 1H NMR DMSO
ppm
1.00
12.25
1.02
0.95
1.01
1.00
1.04
1.00
3.03
BRUKER

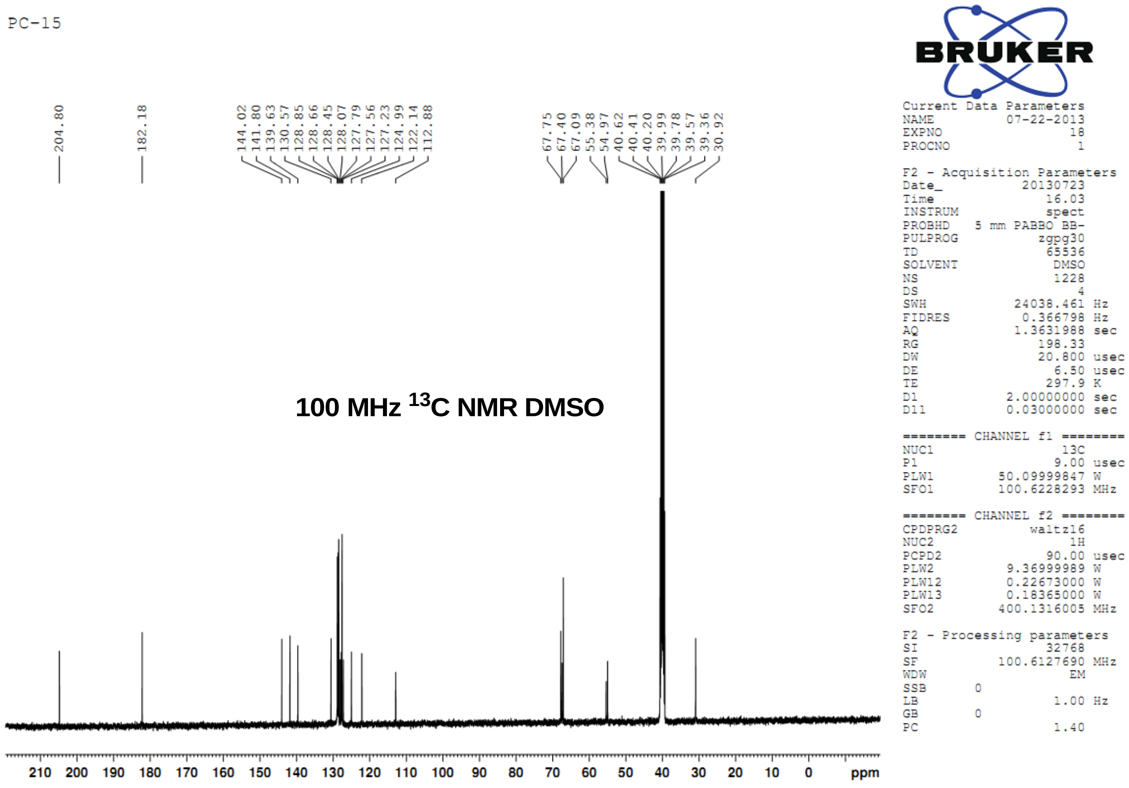

PC-15
204.80
182.18
144.02
141.80
139.63
130.57
128.85
128.66
128.45
128.07
127.79
127.56
127.23
124.99
122.14
112.88
67.75
67.40
67.09
55.38
54.97
40.62
40.41
40.20
39.99
39.78
39.57
39.36
30.92
100 MHz 13C NMR DMSO
ppm
BRUKER

PC-8

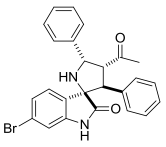

5e

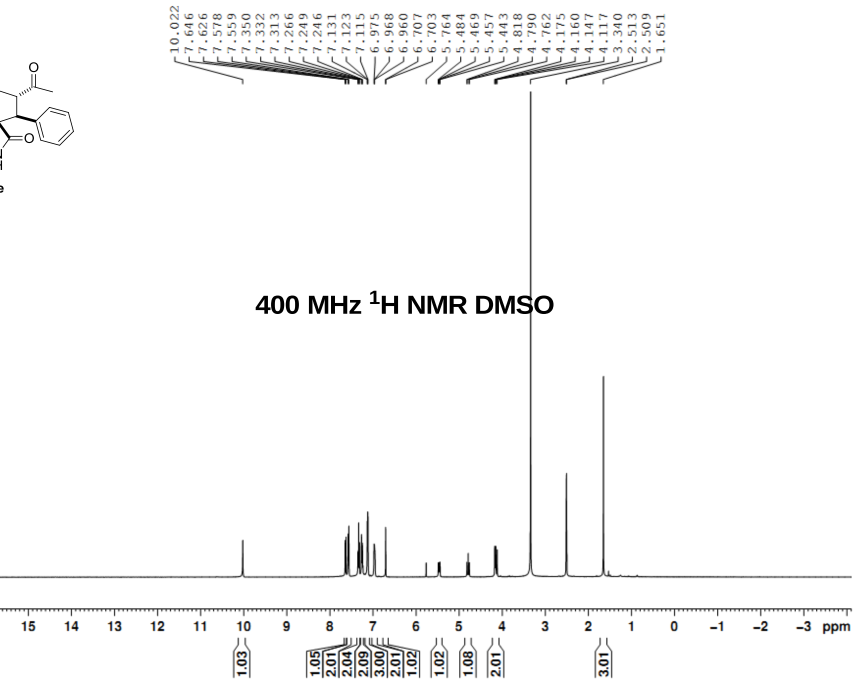

BRUKER

```
Current Data Parameters
NAME          07-22-2013
EXPNO                  4
PROCNO                 1

F2 - Acquisition Parameters
Date_           20130722
Time               15.37
INSTRUM            spect
PROBHD   5 mm PABBO BB-
PULPROG             zg30
TD                 65536
SOLVENT             DMSO
NS                    16
DS                     2
SWH             8223.685 Hz
FIDRES          0.125483 Hz
AQ             3.9846387 sec
RG                198.33
DW                60.800 usec
DE                  6.50 usec
TE                 297.3 K
D1            1.00000000 sec

======== CHANNEL f1 ========
NUC1                  1H
P1                 14.00 usec
PLW1          9.72999954 W
SFO1         400.1324710 MHz

F2 - Processing parameters
SI                 65536
SF           400.1300000 MHz
WDW                   EM
SSB      0
LB                  0.30 Hz
GB       0
PC                  1.00
```

PC-8

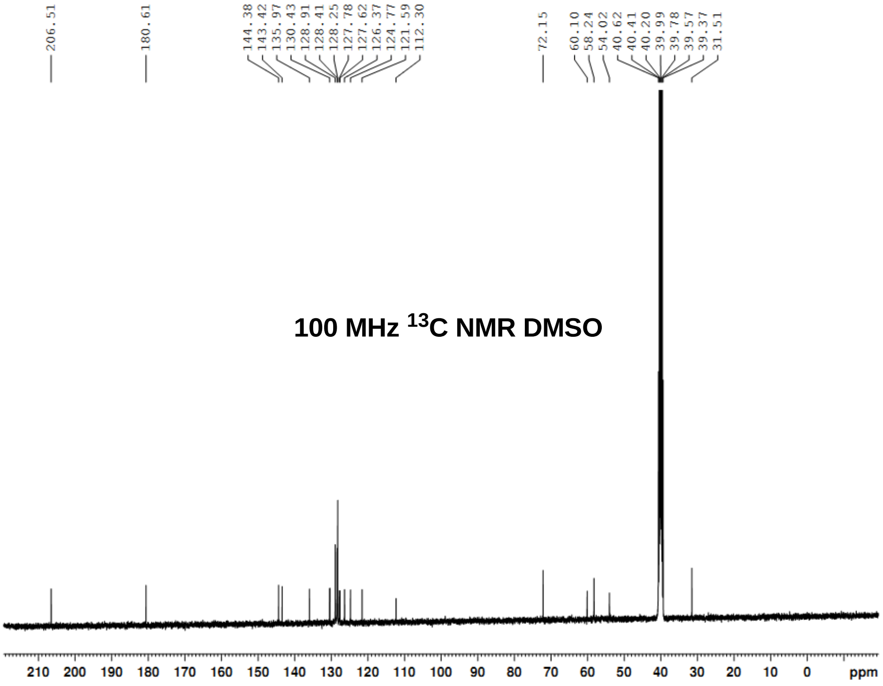

BRUKER

```
Current Data Parameters
NAME          07-22-2013
EXPNO                 19
PROCNO                 1

F2 - Acquisition Parameters
Date_           20130724
Time               14.51
INSTRUM            spect
PROBHD   5 mm PABBO BB-
PULPROG           zgpg30
TD                 65536
SOLVENT             DMSO
NS                  1992
DS                     4
SWH            24038.461 Hz
FIDRES          0.366798 Hz
AQ             1.3631988 sec
RG                168.03
DW                20.800 usec
DE                  6.50 usec
TE                 298.4 K
D1            2.00000000 sec
D11           0.03000000 sec

======== CHANNEL f1 ========
NUC1                 13C
P1                  9.00 usec
PLW1         50.09999847 W
SFO1         100.6228293 MHz

======== CHANNEL f2 ========
CPDPRG2          waltz16
NUC2                  1H
PCPD2              90.00 usec
PLW2          9.36999989 W
PLW12         0.22673000 W
PLW13         0.18365000 W
SFO2         400.1316005 MHz

F2 - Processing parameters
SI                 32768
SF           100.6127690 MHz
WDW                   EM
SSB      0
LB                  1.00 Hz
GB       0
PC                  1.40
```

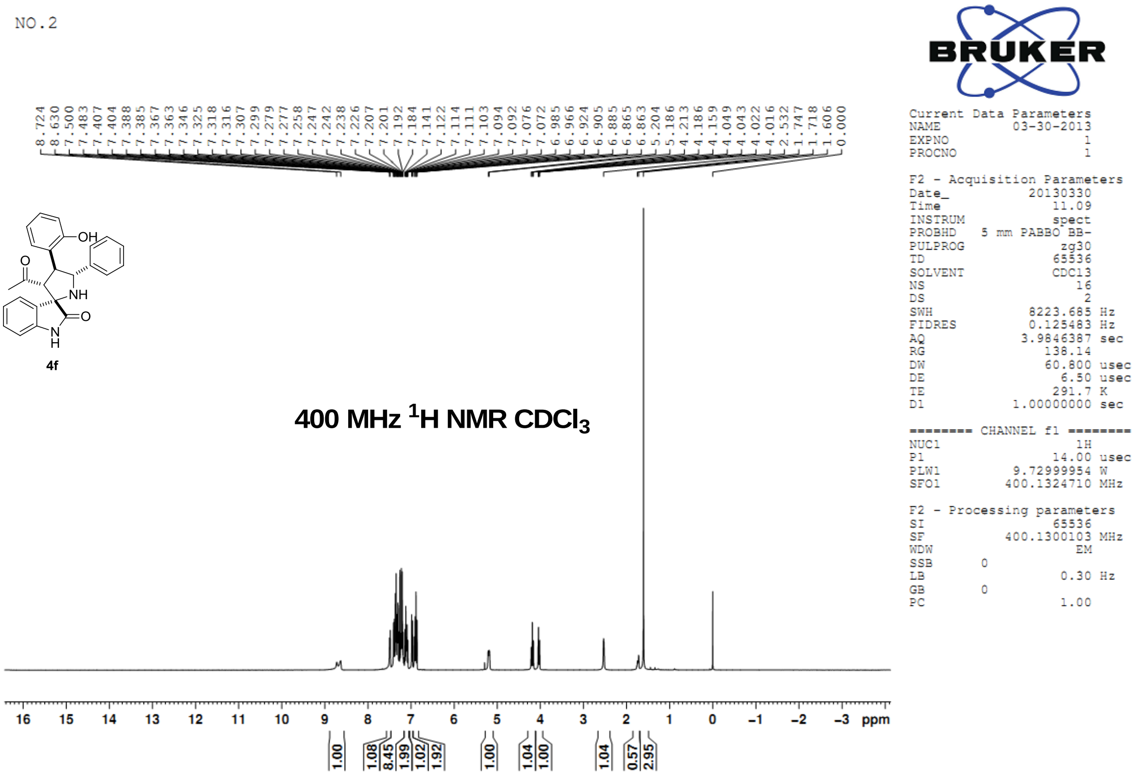
NO.2
8.724
8.630
7.500
7.483
7.407
7.404
7.388
7.385
7.367
7.363
7.346
7.325
7.318
7.316
7.307
7.299
7.279
7.277
7.258
7.247
7.242
7.238
7.226
7.207
7.201
7.192
7.184
7.141
7.122
7.114
7.111
7.103
7.094
7.092
7.076
7.072
6.985
6.966
6.924
6.905
6.885
6.865
6.863
5.204
5.186
4.213
4.186
4.159
4.049
4.043
4.022
4.016
2.532
1.747
1.718
1.606
0.000
OH
O
NH
O
N
H
4f
400 MHz ^{1}H NMR $CDCl_3$
16 15 14 13 12 11 10 9 8 7 6 5 4 3 2 1 0 −1 −2 −3 ppm
1.00
1.08
8.45
1.99
1.02
1.92
1.00
1.04
1.00
1.04
0.57
2.95
BRUKER
Current Data Parameters
NAME 03-30-2013
EXPNO 1
PROCNO 1
F2 - Acquisition Parameters
Date_ 20130330
Time 11.09
INSTRUM spect
PROBHD 5 mm PABBO BB-
PULPROG zg30
TD 65536
SOLVENT CDCl3
NS 16
DS 2
SWH 8223.685 Hz
FIDRES 0.125483 Hz
AQ 3.9846387 sec
RG 138.14
DW 60.800 usec
DE 6.50 usec
TE 291.7 K
D1 1.00000000 sec
======== CHANNEL f1 ========
NUC1 1H
P1 14.00 usec
PLW1 9.72999954 W
SFO1 400.1324710 MHz
F2 - Processing parameters
SI 65536
SF 400.1300103 MHz
WDW EM
SSB 0
LB 0.30 Hz
GB 0
PC 1.00

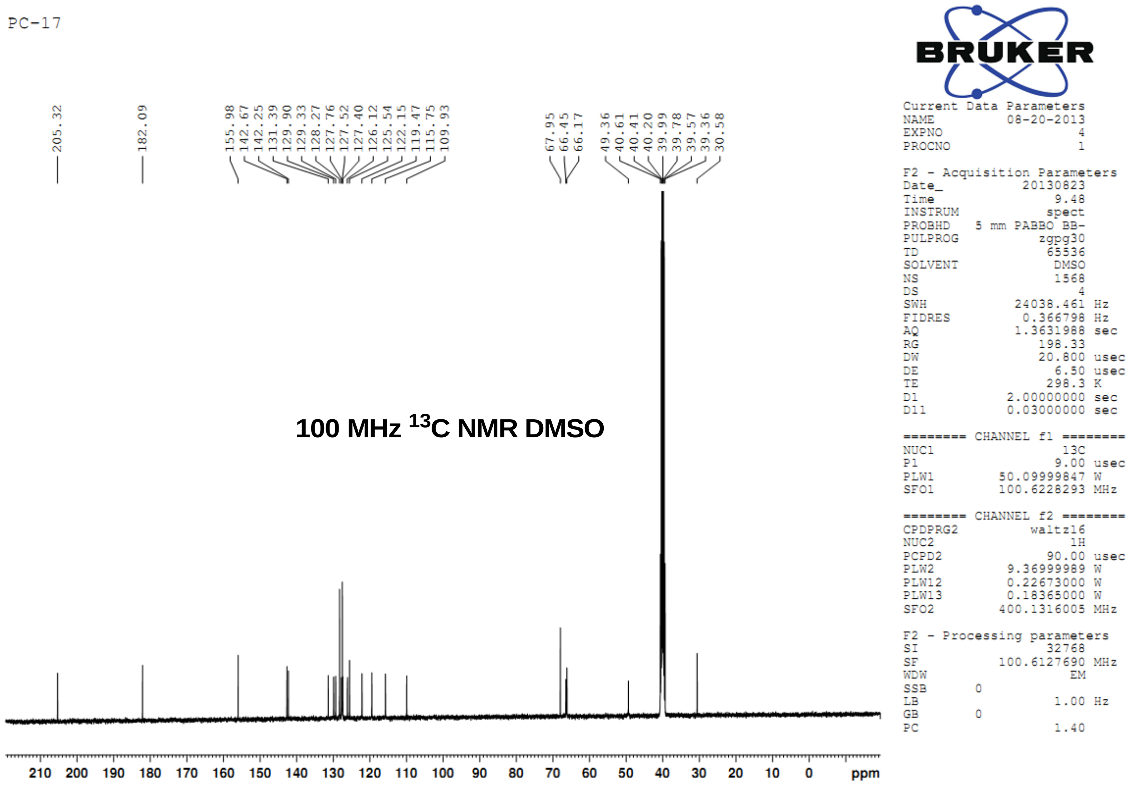
PC-17
205.32
182.09
155.98
142.67
142.25
131.39
129.90
129.33
128.27
127.76
127.52
127.40
126.12
125.54
122.15
119.47
115.75
109.93
67.95
66.45
66.17
49.36
40.61
40.41
40.20
39.99
39.78
39.57
39.36
30.58
100 MHz ^{13}C NMR DMSO
210 200 190 180 170 160 150 140 130 120 110 100 90 80 70 60 50 40 30 20 10 0 ppm
BRUKER
Current Data Parameters
NAME 08-20-2013
EXPNO 4
PROCNO 1
F2 - Acquisition Parameters
Date_ 20130823
Time 9.48
INSTRUM spect
PROBHD 5 mm PABBO BB-
PULPROG zgpg30
TD 65536
SOLVENT DMSO
NS 1568
DS 4
SWH 24038.461 Hz
FIDRES 0.366798 Hz
AQ 1.3631988 sec
RG 198.33
DW 20.800 usec
DE 6.50 usec
TE 298.3 K
D1 2.00000000 sec
D11 0.03000000 sec
======== CHANNEL f1 ========
NUC1 13C
P1 9.00 usec
PLW1 50.09999847 W
SFO1 100.6228293 MHz
======== CHANNEL f2 ========
CPDPRG2 waltz16
NUC2 1H
PCPD2 90.00 usec
PLW2 9.36999989 W
PLW12 0.22673000 W
PLW13 0.18365000 W
SFO2 400.1316005 MHz
F2 - Processing parameters
SI 32768
SF 100.6127690 MHz
WDW EM
SSB 0
LB 1.00 Hz
GB 0
PC 1.40

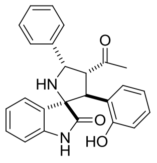

5f

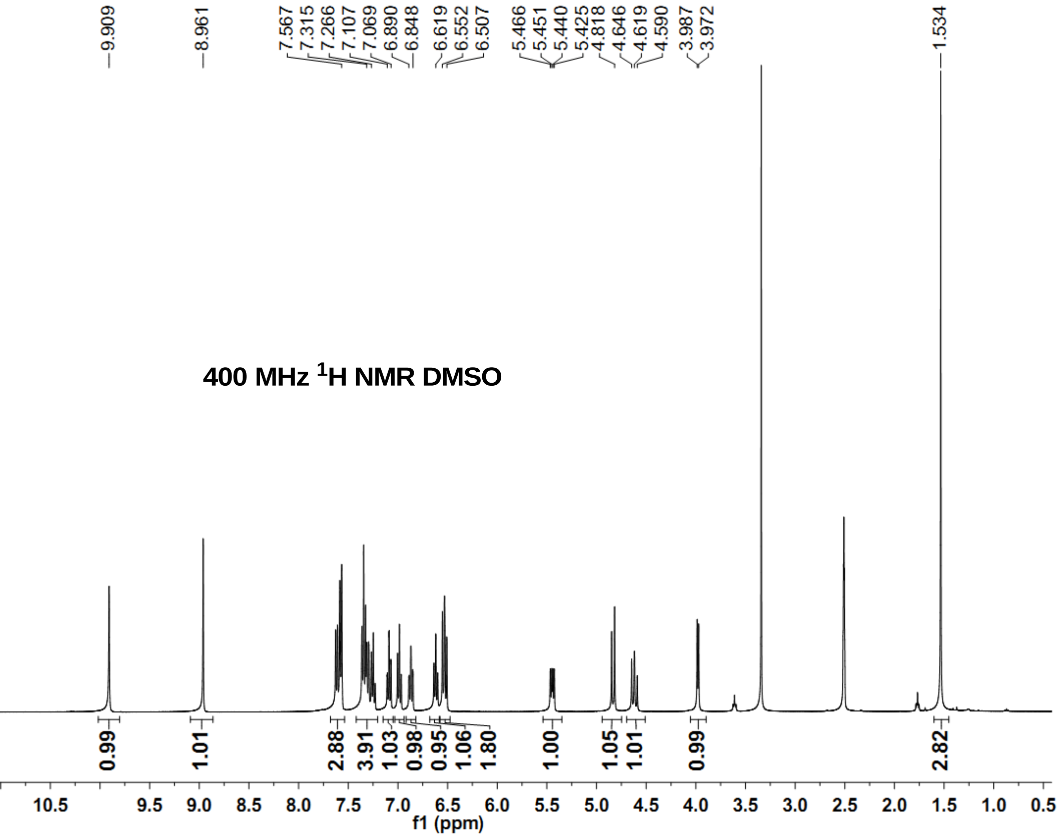

9.909
8.961
7.567
7.315
7.266
7.107
7.069
6.890
6.848
6.619
6.552
6.507
5.466
5.451
5.440
5.425
4.818
4.646
4.619
4.590
3.987
3.972
1.534
400 MHz 1H NMR DMSO
0.99
1.01
2.88
3.91
1.03
0.98
0.95
1.06
1.80
1.00
1.05
1.01
0.99
2.82
12.5
11.5
10.5
9.5
9.0
8.5
8.0
7.5
7.0
6.5
6.0
5.5
5.0
4.5
4.0
3.5
3.0
2.5
2.0
1.5
1.0
0.5
f1 (ppm)

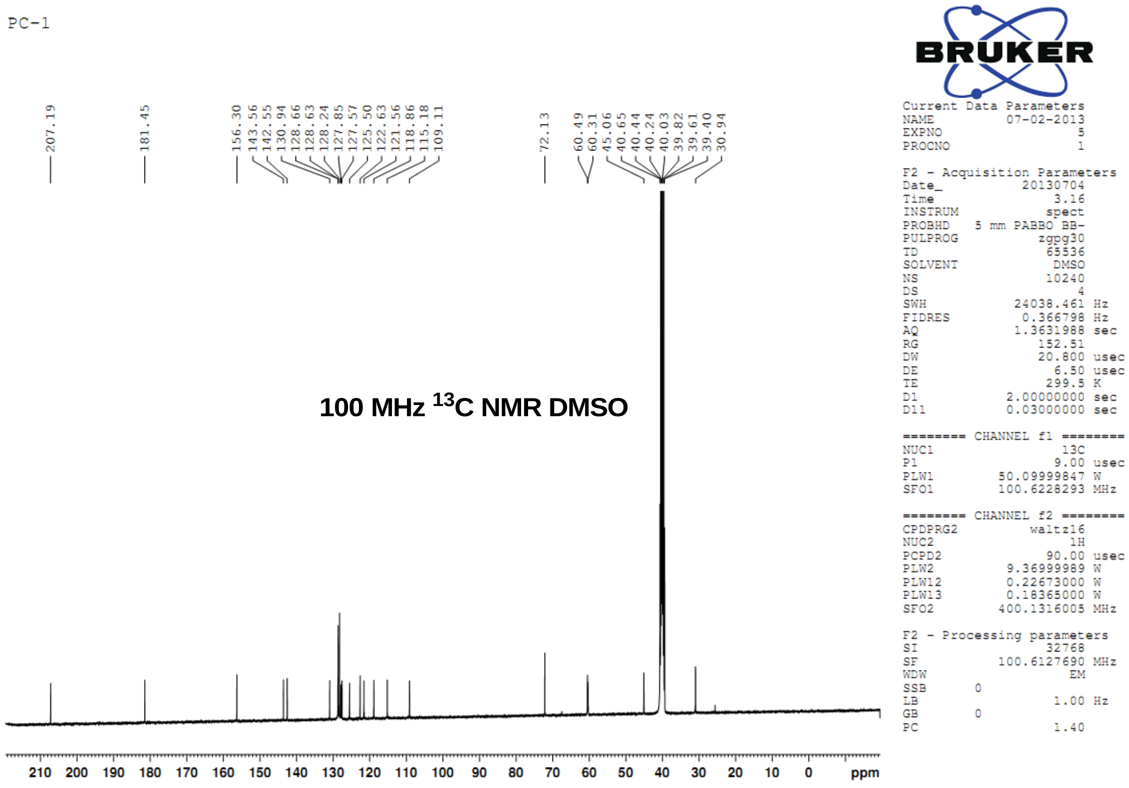

PC-1
207.19
181.45
156.30
143.56
142.55
130.94
128.66
128.63
128.24
127.85
127.57
125.50
122.63
121.56
118.86
115.18
109.11
72.13
60.49
60.31
45.06
40.65
40.44
40.24
40.03
39.82
39.61
39.40
30.94
100 MHz 13C NMR DMSO
210
200
190
180
170
160
150
140
130
120
110
100
90
80
70
60
50
40
30
20
10
0
ppm
BRUKER
Current Data Parameters
NAME 07-02-2013
EXPNO 5
PROCNO 1
F2 - Acquisition Parameters
Date_ 20130704
Time 3.16
INSTRUM spect
PROBHD 5 mm PABBO BB-
PULPROG zgpg30
TD 65536
SOLVENT DMSO
NS 10240
DS 4
SWH 24038.461 Hz
FIDRES 0.366798 Hz
AQ 1.3631988 sec
RG 152.51
DW 20.800 usec
DE 6.50 usec
TE 299.5 K
D1 2.00000000 sec
D11 0.03000000 sec
======== CHANNEL f1 ========
NUC1 13C
P1 9.00 usec
PLW1 50.09999847 W
SFO1 100.6228293 MHz
======== CHANNEL f2 ========
CPDPRG2 waltz16
NUC2 1H
PCPD2 90.00 usec
PLW2 9.36999989 W
PLW12 0.22673000 W
PLW13 0.18365000 W
SFO2 400.1316005 MHz
F2 - Processing parameters
SI 32768
SF 100.6127690 MHz
WDW EM
SSB 0
LB 1.00 Hz
GB 0
PC 1.40

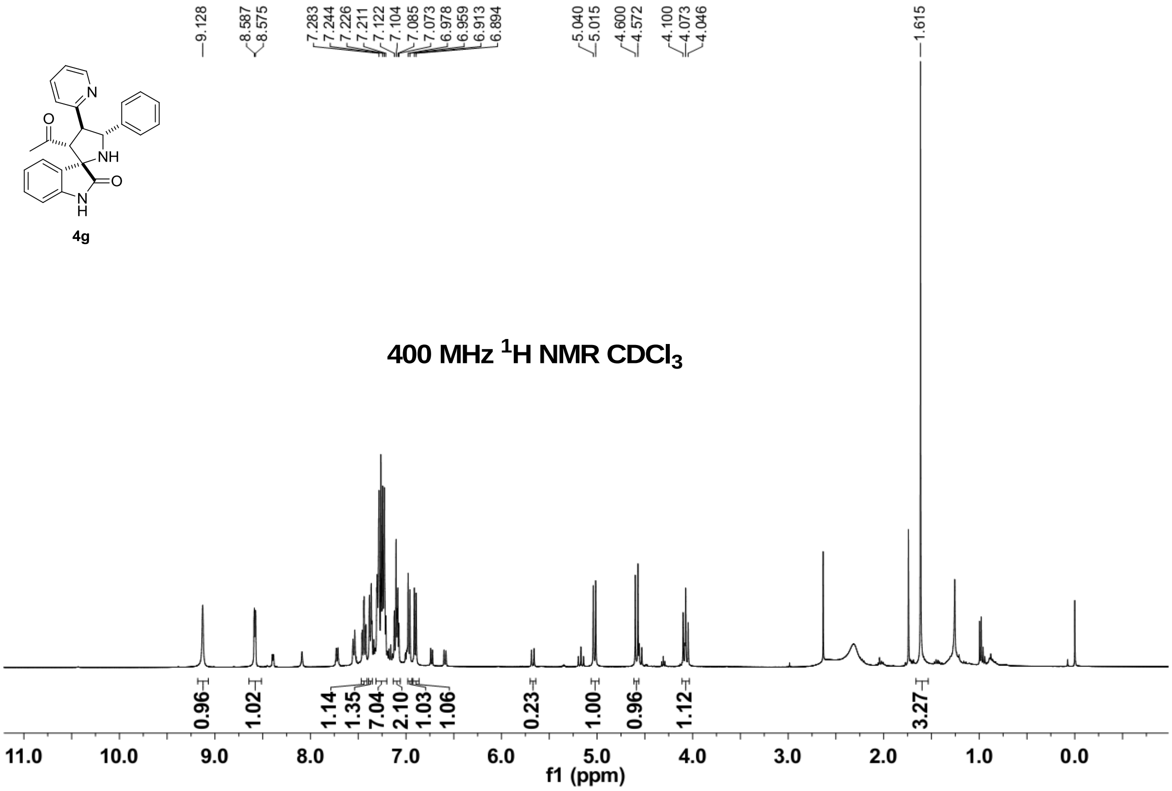
4g
400 MHz ^{1}H NMR $CDCl_3$
9.128
8.587
8.575
7.283
7.244
7.226
7.211
7.122
7.104
7.085
7.073
6.978
6.959
6.913
6.894
5.040
5.015
4.600
4.572
4.100
4.073
4.046
1.615
0.96
1.02
1.14
1.35
7.04
2.10
1.03
1.06
0.23
1.00
0.96
1.12
3.27
11.0
10.0
9.0
8.0
7.0
6.0
5.0
4.0
3.0
2.0
1.0
0.0
f1 (ppm)

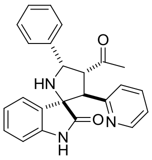

5g

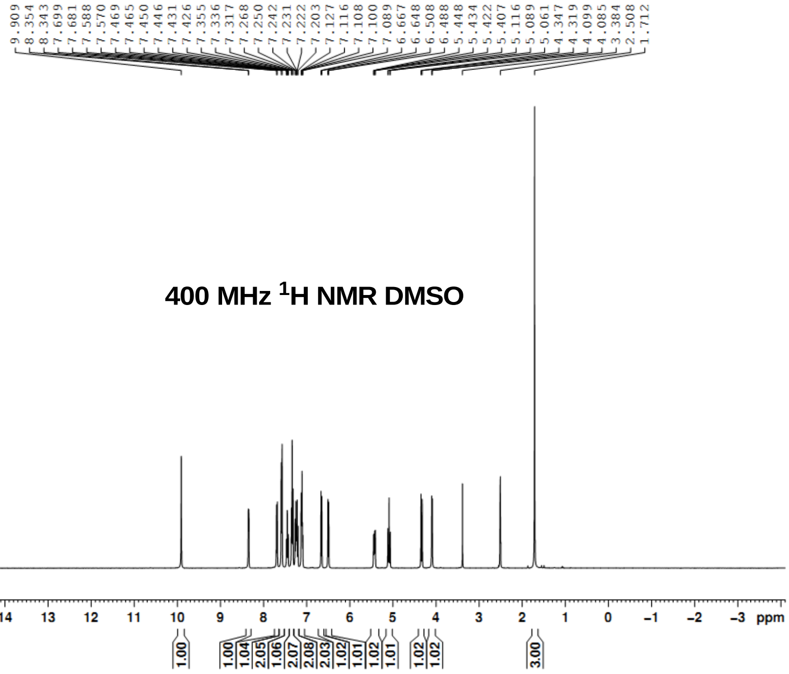


```
Current Data Parameters
NAME            04-08-2013
EXPNO                    3
PROCNO                   1

F2 - Acquisition Parameters
Date_             20130408
Time                 13.57
INSTRUM              spect
PROBHD   5 mm PABBO BB-
PULPROG               zg30
TD                   65536
SOLVENT               DMSO
NS                      16
DS                       2
SWH               8223.685 Hz
FIDRES            0.125483 Hz
AQ               3.9846387 sec
RG                   87.39
DW                  60.800 usec
DE                    6.50 usec
TE                   290.0 K
D1              1.00000000 sec

======== CHANNEL f1 ========
NUC1                    1H
P1                   14.00 usec
PLW1            9.72999954 W
SFO1           400.1324710 MHz

F2 - Processing parameters
SI                   65536
SF             400.1300000 MHz
WDW                     EM
SSB      0
LB                    0.30 Hz
GB       0
PC                    1.00
```

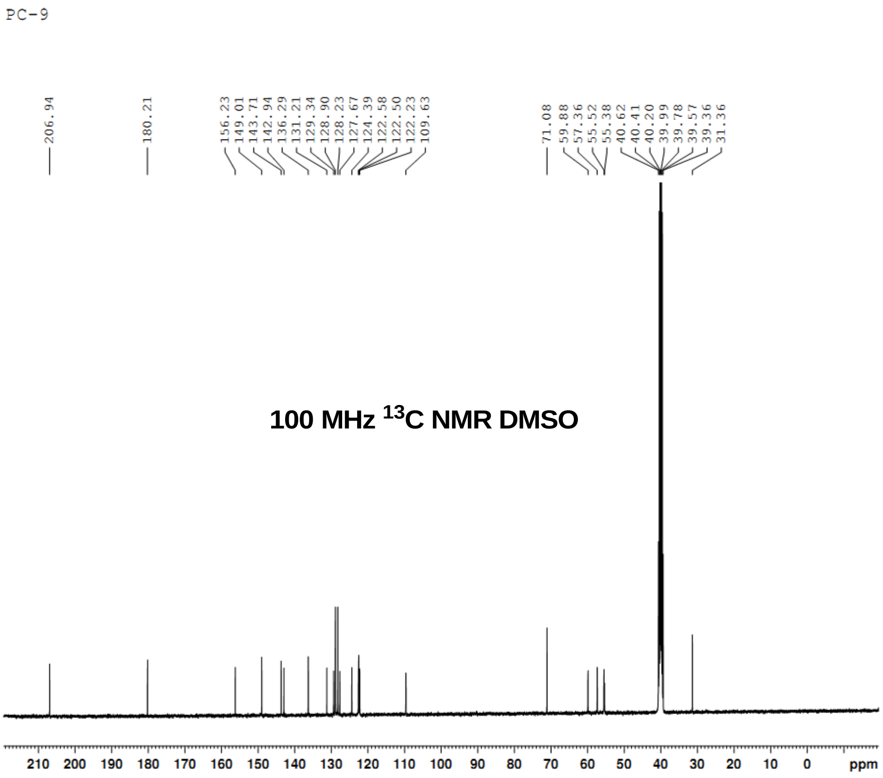


```
Current Data Parameters
NAME            07-22-2013
EXPNO                   14
PROCNO                   1

F2 - Acquisition Parameters
Date_             20130724
Time                  1.47
INSTRUM              spect
PROBHD   5 mm PABBO BB-
PULPROG             zgpg30
TD                   65536
SOLVENT               DMSO
NS                    3072
DS                       4
SWH              24038.461 Hz
FIDRES            0.366798 Hz
AQ               1.3631988 sec
RG                  198.33
DW                  20.800 usec
DE                    6.50 usec
TE                   298.2 K
D1              2.00000000 sec
D11             0.03000000 sec

======== CHANNEL f1 ========
NUC1                   13C
P1                    9.00 usec
PLW1           50.09999847 W
SFO1           100.6228293 MHz

======== CHANNEL f2 ========
CPDPRG2            waltz16
NUC2                    1H
PCPD2                90.00 usec
PLW2            9.36999989 W
PLW12           0.22673000 W
PLW13           0.18365000 W
SFO2           400.1316005 MHz

F2 - Processing parameters
SI                   32768
SF             100.6127690 MHz
WDW                     EM
SSB      0
LB                    1.00 Hz
GB       0
PC                    1.40
```

NO.5

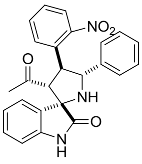

4h

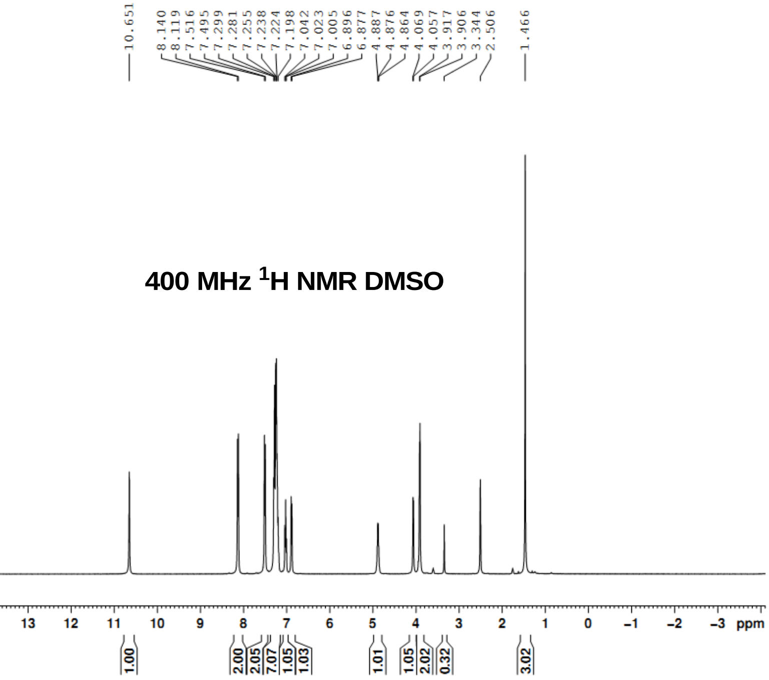

Current Data Parameters
NAME 03-30-2013
EXPNO 5
PROCNO 1

F2 - Acquisition Parameters
Date_ 20130330
Time 14.28
INSTRUM spect
PROBHD 5 mm PABBO BB-
PULPROG zg30
TD 65536
SOLVENT DMSO
NS 16
DS 2
SWH 8223.685 Hz
FIDRES 0.125483 Hz
AQ 3.9846387 sec
RG 94.96
DW 60.800 usec
DE 6.50 usec
TE 297.8 K
D1 1.00000000 sec

======== CHANNEL f1 ========
NUC1 1H
P1 14.00 usec
PLW1 9.72999954 W
SFO1 400.1324710 MHz

F2 - Processing parameters
SI 65536
SF 400.1300000 MHz
WDW EM
SSB 0
LB 0.30 Hz
GB 0
PC 1.00

NO.5

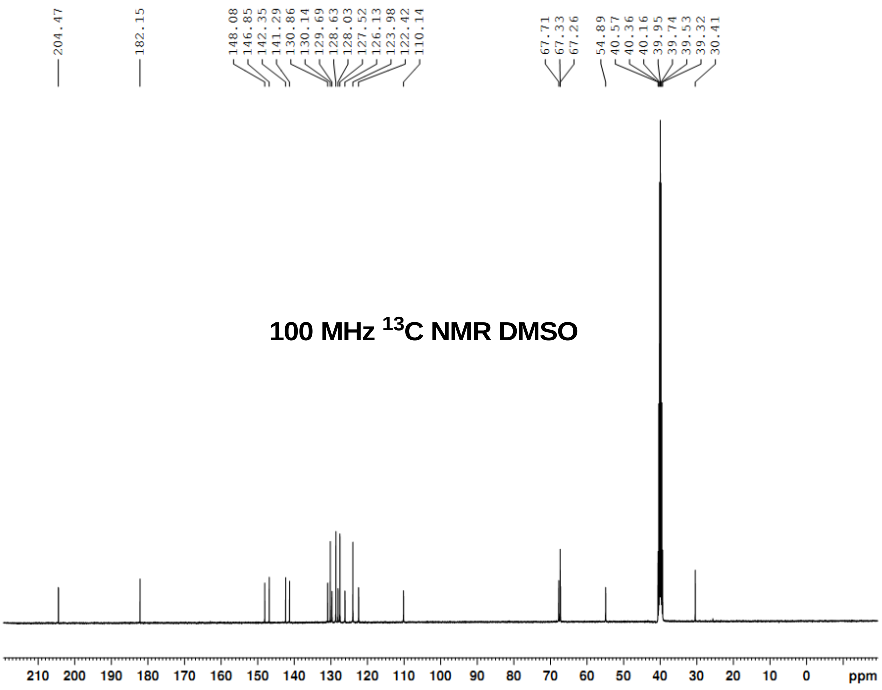

Current Data Parameters
NAME 03-31-2013
EXPNO 3
PROCNO 1

F2 - Acquisition Parameters
Date_ 20130331
Time 0.36
INSTRUM spect
PROBHD 5 mm PABBO BB-
PULPROG zgpg30
TD 65536
SOLVENT DMSO
NS 2048
DS 4
SWH 24038.461 Hz
FIDRES 0.366798 Hz
AQ 1.3631988 sec
RG 198.33
DW 20.800 usec
DE 6.50 usec
TE 292.5 K
D1 2.00000000 sec
D11 0.03000000 sec

======== CHANNEL f1 ========
NUC1 13C
P1 9.00 usec
PLW1 50.09999847 W
SFO1 100.6228293 MHz

======== CHANNEL f2 ========
CPDPRG2 waltz16
NUC2 1H
PCPD2 90.00 usec
PLW2 9.36999989 W
PLW12 0.22673000 W
PLW13 0.18365000 W
SFO2 400.1316005 MHz

F2 - Processing parameters
SI 32768
SF 100.6127690 MHz
WDW EM
SSB 0
LB 1.00 Hz
GB 0
PC 1.40

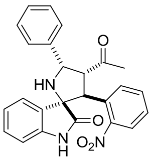

5h

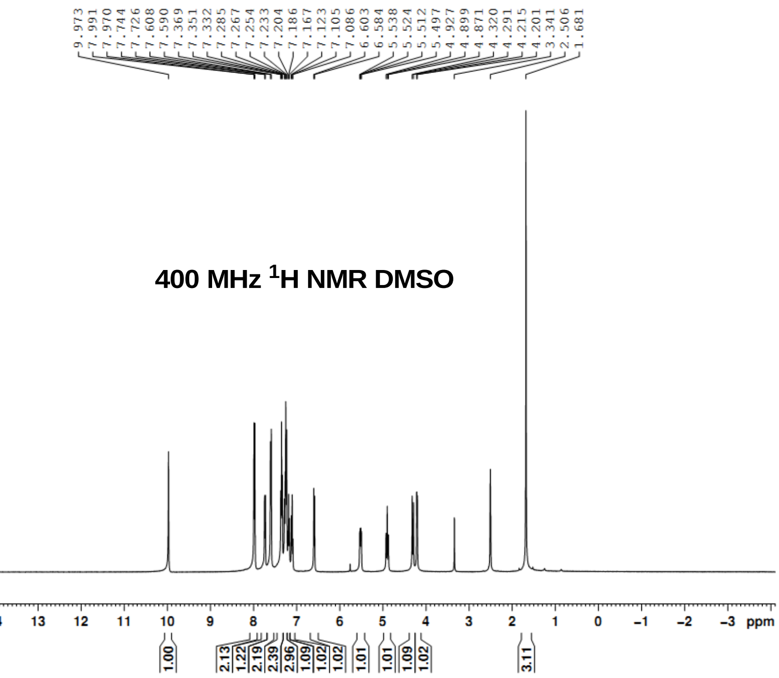

BRUKER

```
Current Data Parameters
NAME           03-30-2013
EXPNO                   4
PROCNO                  1

F2 - Acquisition Parameters
Date_            20130330
Time                14.23
INSTRUM             spect
PROBHD   5 mm PABBO BB-
PULPROG              zg30
TD                  65536
SOLVENT              DMSO
NS                     16
DS                      2
SWH              8223.685 Hz
FIDRES           0.125483 Hz
AQ              3.9846387 sec
RG                  94.96
DW                 60.800 usec
DE                   6.50 usec
TE                  297.9 K
D1             1.00000000 sec

======== CHANNEL f1 ========
NUC1                   1H
P1                  14.00 usec
PLW1           9.72999954 W
SFO1          400.1324710 MHz

F2 - Processing parameters
SI                  65536
SF            400.1300000 MHz
WDW                    EM
SSB      0
LB                   0.30 Hz
GB       0
PC                   1.00
```

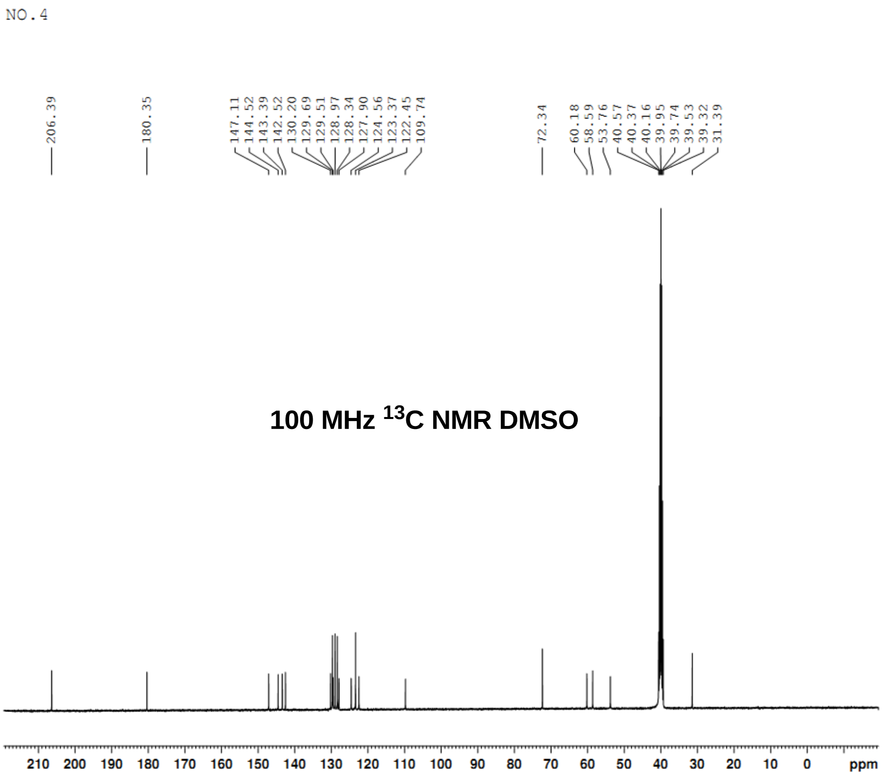

BRUKER

```
Current Data Parameters
NAME           03-31-2013
EXPNO                   2
PROCNO                  1

F2 - Acquisition Parameters
Date_            20130330
Time                22.36
INSTRUM             spect
PROBHD   5 mm PABBO BB-
PULPROG            zgpg30
TD                  65536
SOLVENT              DMSO
NS                   2048
DS                      4
SWH             24038.461 Hz
FIDRES           0.366798 Hz
AQ              1.3631988 sec
RG                 198.33
DW                 20.800 usec
DE                   6.50 usec
TE                  292.8 K
D1             2.00000000 sec
D11            0.03000000 sec

======== CHANNEL f1 ========
NUC1                  13C
P1                   9.00 usec
PLW1          50.09999847 W
SFO1          100.6228293 MHz

======== CHANNEL f2 ========
CPDPRG2           waltz16
NUC2                   1H
PCPD2               90.00 usec
PLW2           9.36999989 W
PLW12          0.22673000 W
PLW13          0.18365000 W
SFO2          400.1316005 MHz

F2 - Processing parameters
SI                  32768
SF            100.6127690 MHz
WDW                    EM
SSB      0
LB                   1.00 Hz
GB       0
PC                   1.40
```

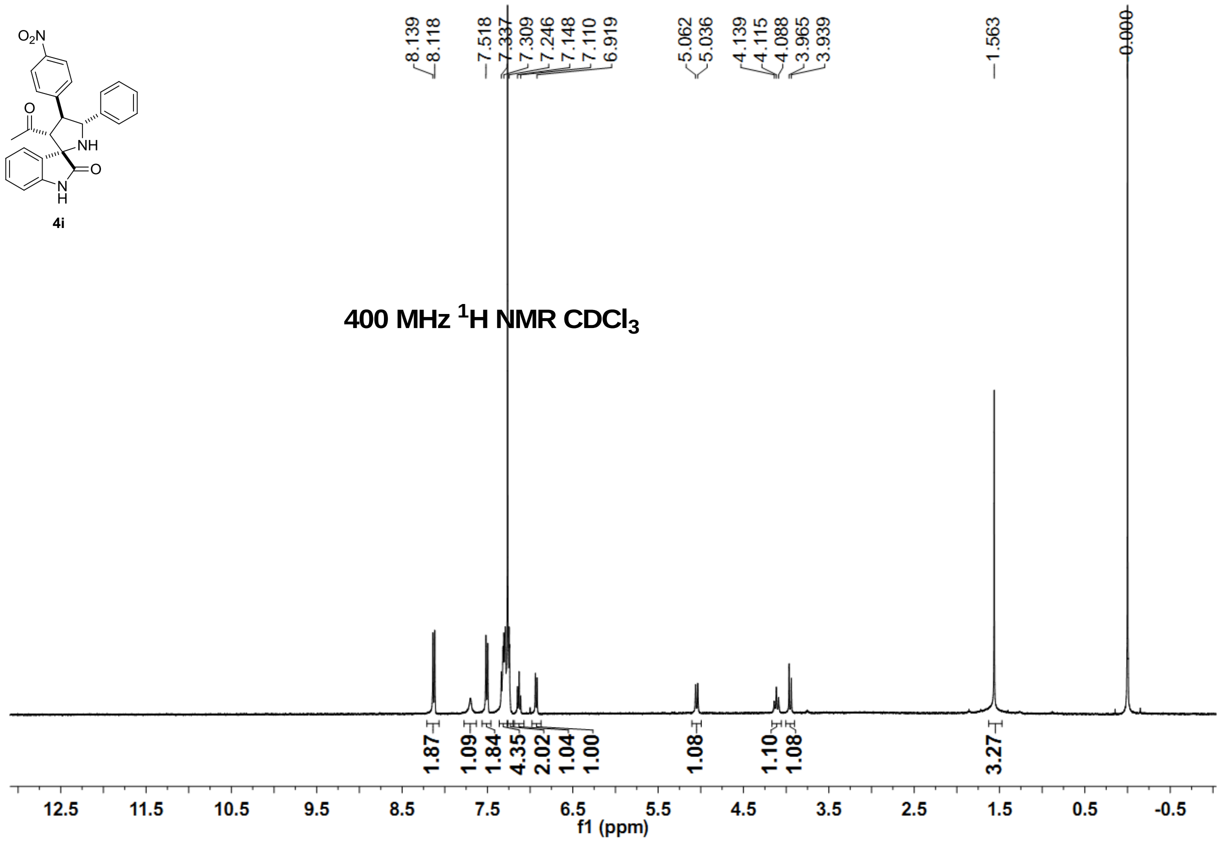
4i
400 MHz ^{1}H NMR $CDCl_3$
f1 (ppm)

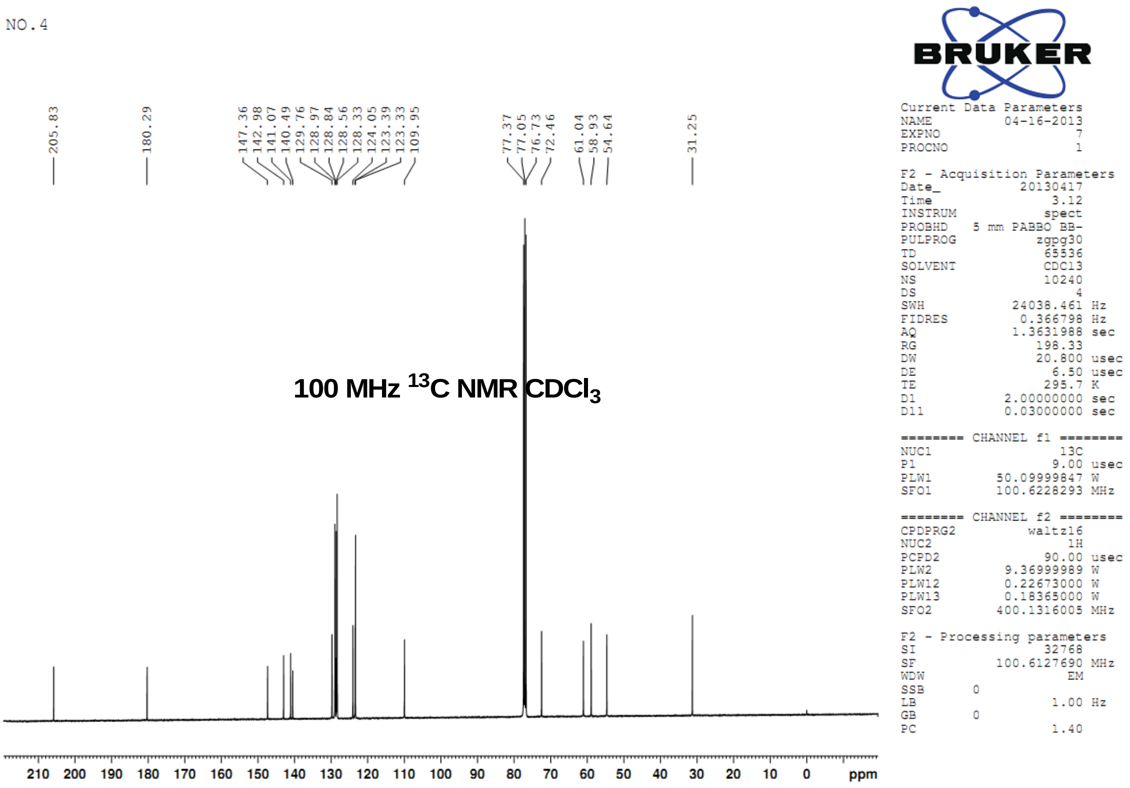
NO.4
100 MHz ^{13}C NMR $CDCl_3$
ppm
BRUKER
Current Data Parameters
NAME 04-16-2013
EXPNO 7
PROCNO 1
F2 - Acquisition Parameters
Date_ 20130417
Time 3.12
INSTRUM spect
PROBHD 5 mm PABBO BB-
PULPROG zgpg30
TD 65536
SOLVENT CDCl3
NS 10240
DS 4
SWH 24038.461 Hz
FIDRES 0.366798 Hz
AQ 1.3631988 sec
RG 198.33
DW 20.800 usec
DE 6.50 usec
TE 295.7 K
D1 2.00000000 sec
D11 0.03000000 sec
======== CHANNEL f1 ========
NUC1 13C
P1 9.00 usec
PLW1 50.09999847 W
SFO1 100.6228293 MHz
======== CHANNEL f2 ========
CPDPRG2 waltz16
NUC2 1H
PCPD2 90.00 usec
PLW2 9.36999989 W
PLW12 0.22673000 W
PLW13 0.18365000 W
SFO2 400.1316005 MHz
F2 - Processing parameters
SI 32768
SF 100.6127690 MHz
WDW EM
SSB 0
LB 1.00 Hz
GB 0
PC 1.40

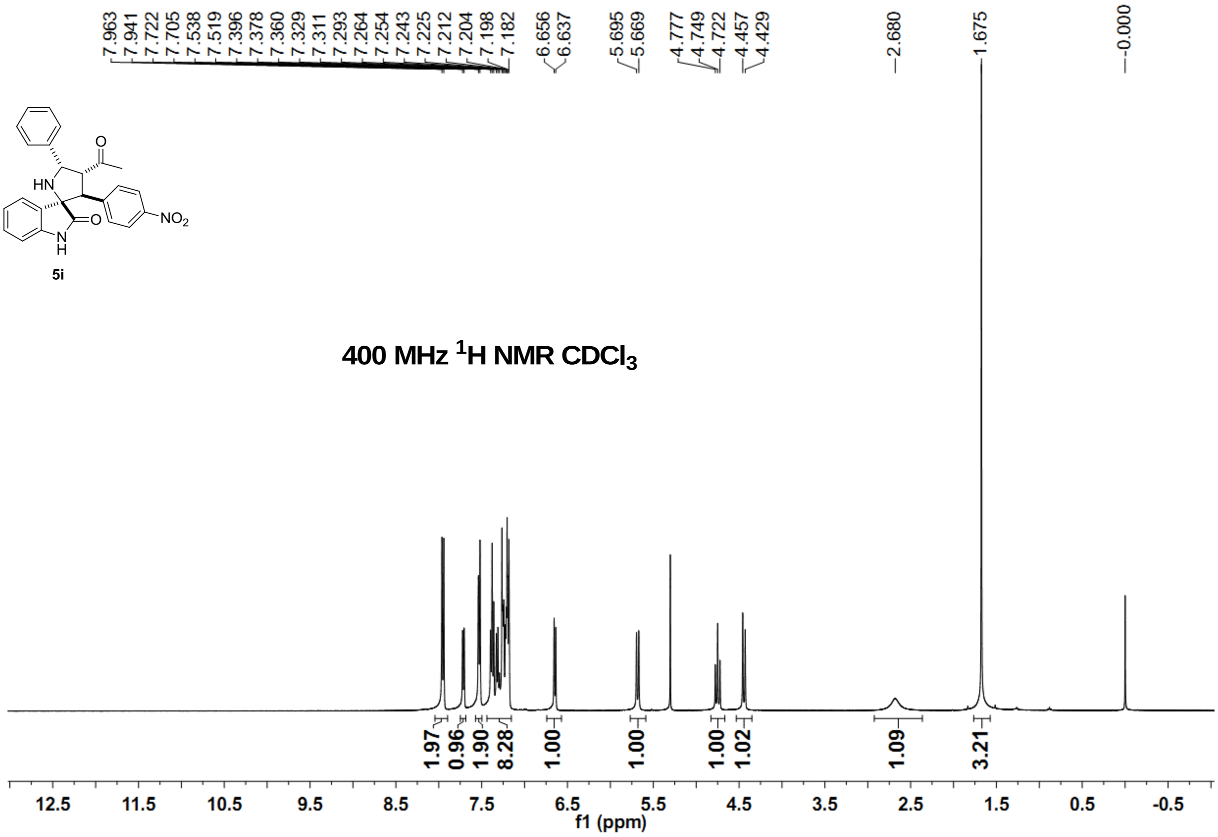

PC-27

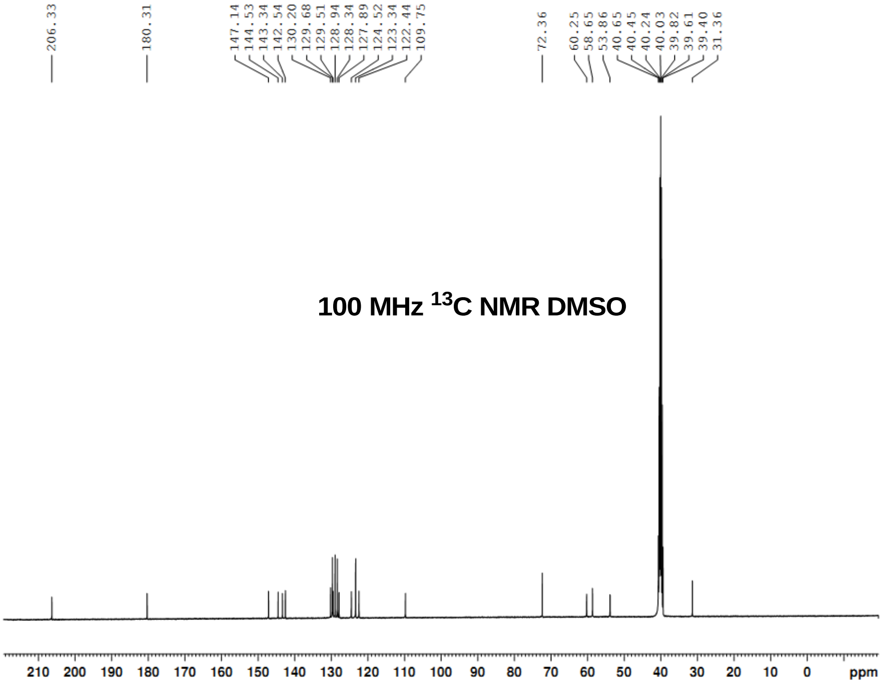

BRUKER

```
Current Data Parameters
NAME              09-04-2013
EXPNO                      6
PROCNO                     1

F2 - Acquisition Parameters
Date_               20130905
Time                    3.36
INSTRUM                spect
PROBHD   5 mm PABBO BB-
PULPROG              zgpg30
TD                     65536
SOLVENT                 DMSO
NS                     10240
DS                         4
SWH             24038.461 Hz
FIDRES           0.366798 Hz
AQ              1.3631988 sec
RG                    198.33
DW                    20.800 usec
DE                      6.50 usec
TE                     299.2 K
D1             2.00000000 sec
D11            0.03000000 sec

======== CHANNEL f1 ========
NUC1                     13C
P1                      9.00 usec
PLW1             50.09999847 W
SFO1           100.6228293 MHz

======== CHANNEL f2 ========
CPDPRG2              waltz16
NUC2                      1H
PCPD2                  90.00 usec
PLW2              9.36999989 W
PLW12             0.22673000 W
PLW13             0.18365000 W
SFO2           400.1316005 MHz

F2 - Processing parameters
SI                     32768
SF             100.6127690 MHz
WDW                       EM
SSB      0
LB                      1.00 Hz
GB       0
PC                      1.40
```

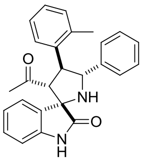

4j

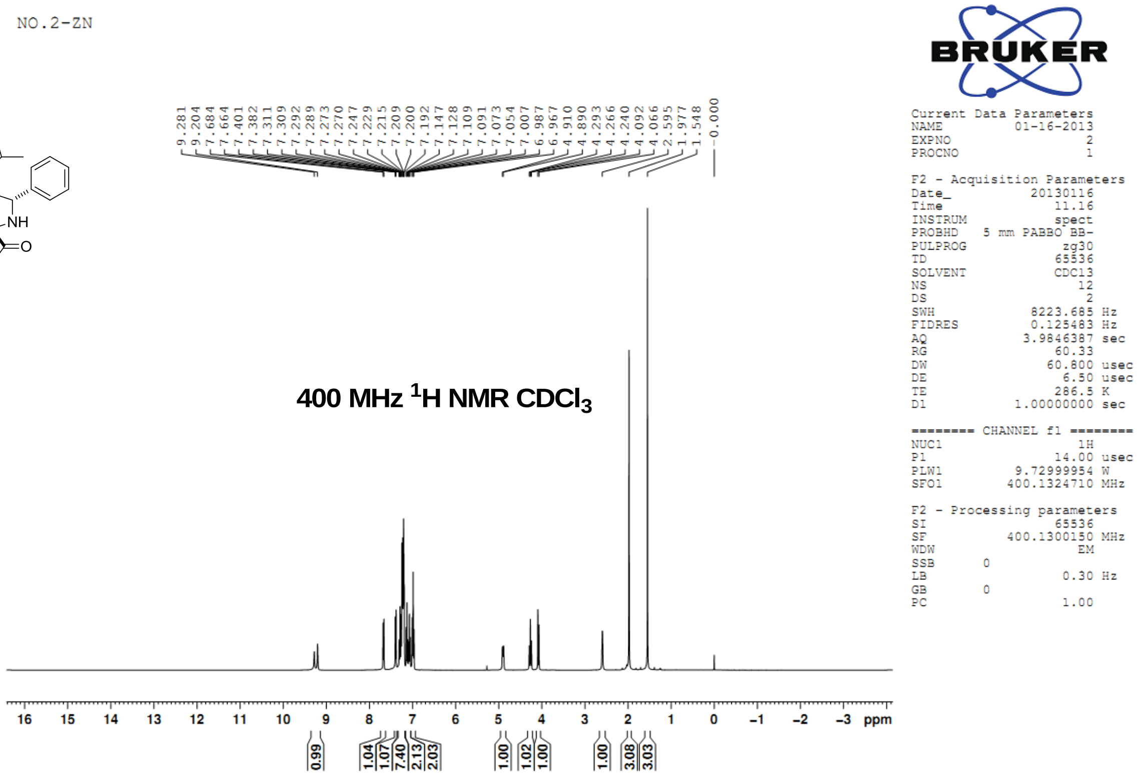
NO.2-ZN
9.281 9.204 7.684 7.664 7.401 7.382 7.311 7.309 7.292 7.289 7.273 7.270 7.247 7.229 7.215 7.209 7.200 7.192 7.147 7.128 7.109 7.091 7.073 7.054 7.007 6.987 6.967 4.910 4.890 4.293 4.266 4.240 4.092 4.066 2.595 1.977 1.548 0.000
400 MHz 1H NMR CDCl3
16 15 14 13 12 11 10 9 8 7 6 5 4 3 2 1 0 -1 -2 -3 ppm
0.99 1.04 1.07 7.40 2.13 2.03 1.00 1.02 1.00 1.00 3.08 3.03
BRUKER
Current Data Parameters
NAME 01-16-2013
EXPNO 2
PROCNO 1
F2 - Acquisition Parameters
Date_ 20130116
Time 11.16
INSTRUM spect
PROBHD 5 mm PABBO BB-
PULPROG zg30
TD 65536
SOLVENT CDCl3
NS 12
DS 2
SWH 8223.685 Hz
FIDRES 0.125483 Hz
AQ 3.9846387 sec
RG 60.33
DW 60.800 usec
DE 6.50 usec
TE 286.5 K
D1 1.00000000 sec
======== CHANNEL f1 ========
NUC1 1H
P1 14.00 usec
PLW1 9.72999954 W
SFO1 400.1324710 MHz
F2 - Processing parameters
SI 65536
SF 400.1300150 MHz
WDW EM
SSB 0
LB 0.30 Hz
GB 0
PC 1.00

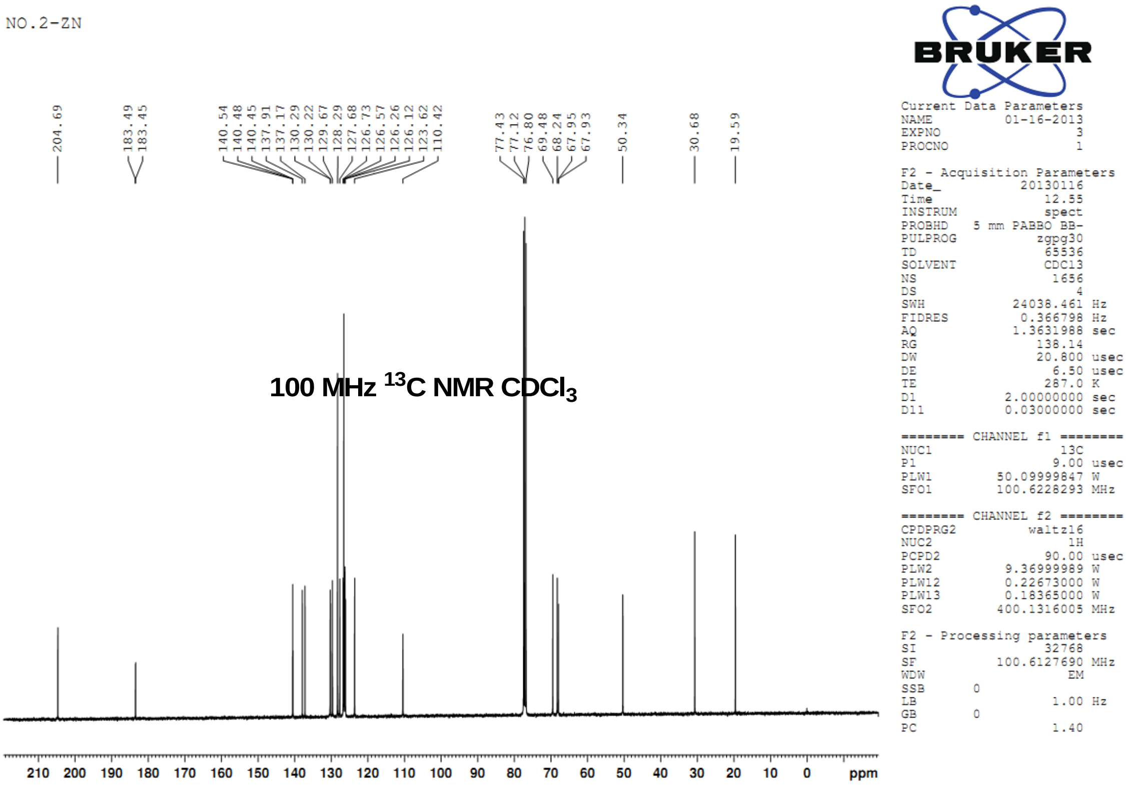
NO.2-ZN
204.69 183.49 183.45 140.54 140.48 140.45 137.91 137.17 130.29 130.22 129.67 128.29 127.68 126.73 126.57 126.26 126.12 123.62 110.42 77.43 77.12 76.80 69.48 68.24 67.95 67.93 50.34 30.68 19.59
100 MHz 13C NMR CDCl3
210 200 190 180 170 160 150 140 130 120 110 100 90 80 70 60 50 40 30 20 10 0 ppm
BRUKER
Current Data Parameters
NAME 01-16-2013
EXPNO 3
PROCNO 1
F2 - Acquisition Parameters
Date_ 20130116
Time 12.55
INSTRUM spect
PROBHD 5 mm PABBO BB-
PULPROG zgpg30
TD 65536
SOLVENT CDCl3
NS 1656
DS 4
SWH 24038.461 Hz
FIDRES 0.366798 Hz
AQ 1.3631988 sec
RG 138.14
DW 20.800 usec
DE 6.50 usec
TE 287.0 K
D1 2.00000000 sec
D11 0.03000000 sec
======== CHANNEL f1 ========
NUC1 13C
P1 9.00 usec
PLW1 50.09999847 W
SFO1 100.6228293 MHz
======== CHANNEL f2 ========
CPDPRG2 waltz16
NUC2 1H
PCPD2 90.00 usec
PLW2 9.36999989 W
PLW12 0.22673000 W
PLW13 0.18365000 W
SFO2 400.1316005 MHz
F2 - Processing parameters
SI 32768
SF 100.6127690 MHz
WDW EM
SSB 0
LB 1.00 Hz
GB 0
PC 1.40

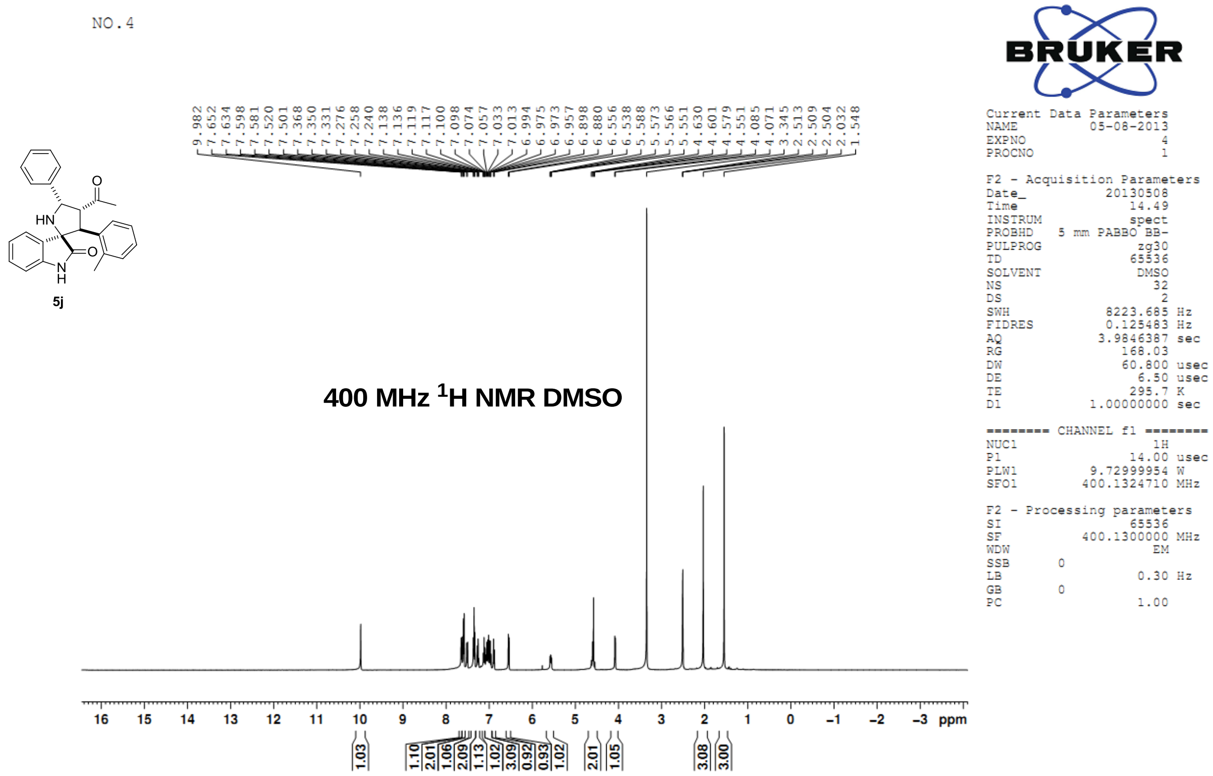
NO.4
5j
400 MHz 1H NMR DMSO
9.982 7.652 7.634 7.598 7.581 7.520 7.501 7.368 7.350 7.331 7.276 7.258 7.240 7.138 7.136 7.119 7.117 7.100 7.098 7.074 7.057 7.033 7.013 6.994 6.975 6.973 6.957 6.898 6.880 6.556 6.538 5.588 5.573 5.566 5.551 4.630 4.601 4.579 4.551 4.085 4.071 3.345 2.513 2.509 2.504 2.032 1.548
1.03 1.10 2.01 1.06 2.09 1.13 1.02 3.09 0.92 0.93 1.02 2.01 1.05 3.08 3.00
16 15 14 13 12 11 10 9 8 7 6 5 4 3 2 1 0 −1 −2 −3 ppm
BRUKER
Current Data Parameters
NAME 05-08-2013
EXPNO 4
PROCNO 1
F2 - Acquisition Parameters
Date_ 20130508
Time 14.49
INSTRUM spect
PROBHD 5 mm PABBO BB-
PULPROG zg30
TD 65536
SOLVENT DMSO
NS 32
DS 2
SWH 8223.685 Hz
FIDRES 0.125483 Hz
AQ 3.9846387 sec
RG 168.03
DW 60.800 usec
DE 6.50 usec
TE 295.7 K
D1 1.00000000 sec
======== CHANNEL f1 ========
NUC1 1H
P1 14.00 usec
PLW1 9.72999954 W
SFO1 400.1324710 MHz
F2 - Processing parameters
SI 65536
SF 400.1300000 MHz
WDW EM
SSB 0
LB 0.30 Hz
GB 0
PC 1.00

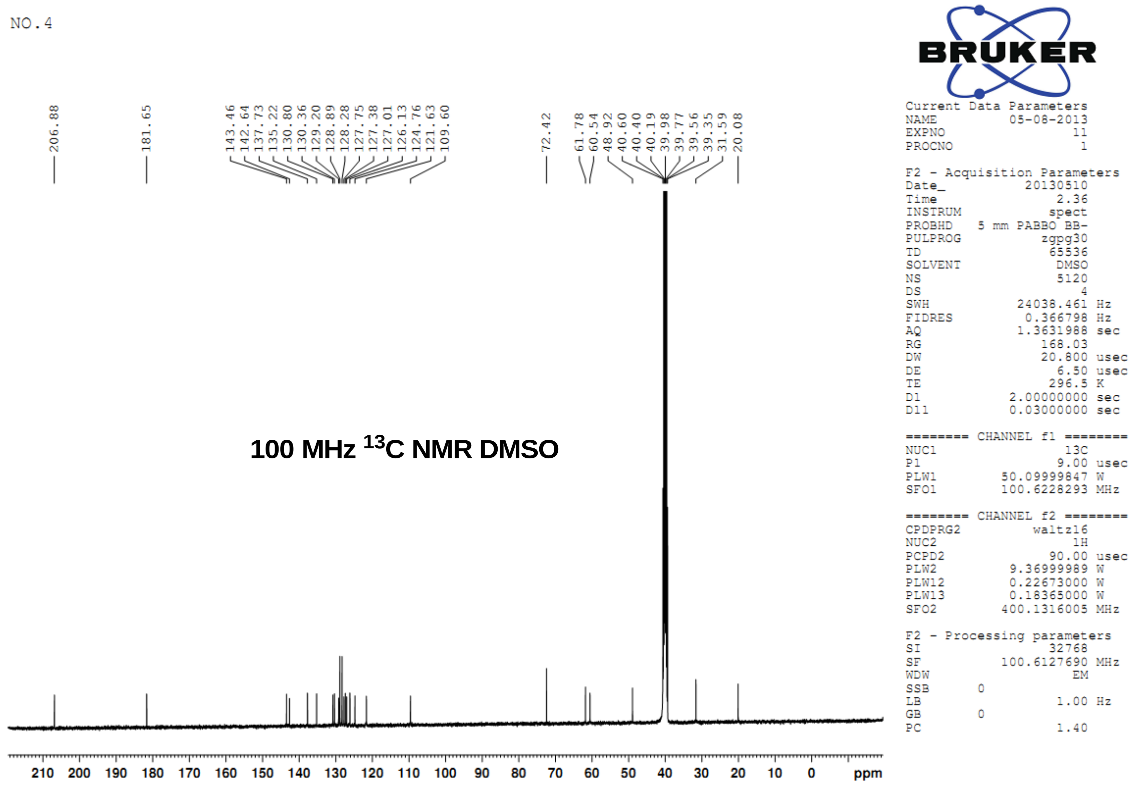
NO.4
206.88 181.65 143.46 142.64 137.73 135.22 130.80 130.36 129.20 128.89 128.28 127.75 127.38 127.01 126.13 124.76 121.63 109.60 72.42 61.78 60.54 48.92 40.60 40.40 40.19 39.98 39.77 39.56 39.35 31.59 20.08
100 MHz 13C NMR DMSO
210 200 190 180 170 160 150 140 130 120 110 100 90 80 70 60 50 40 30 20 10 0 ppm
BRUKER
Current Data Parameters
NAME 05-08-2013
EXPNO 11
PROCNO 1
F2 - Acquisition Parameters
Date_ 20130510
Time 2.36
INSTRUM spect
PROBHD 5 mm PABBO BB-
PULPROG zgpg30
TD 65536
SOLVENT DMSO
NS 5120
DS 4
SWH 24038.461 Hz
FIDRES 0.366798 Hz
AQ 1.3631988 sec
RG 168.03
DW 20.800 usec
DE 6.50 usec
TE 296.5 K
D1 2.00000000 sec
D11 0.03000000 sec
======== CHANNEL f1 ========
NUC1 13C
P1 9.00 usec
PLW1 50.09999847 W
SFO1 100.6228293 MHz
======== CHANNEL f2 ========
CPDPRG2 waltz16
NUC2 1H
PCPD2 90.00 usec
PLW2 9.36999989 W
PLW12 0.22673000 W
PLW13 0.18365000 W
SFO2 400.1316005 MHz
F2 - Processing parameters
SI 32768
SF 100.6127690 MHz
WDW EM
SSB 0
LB 1.00 Hz
GB 0
PC 1.40

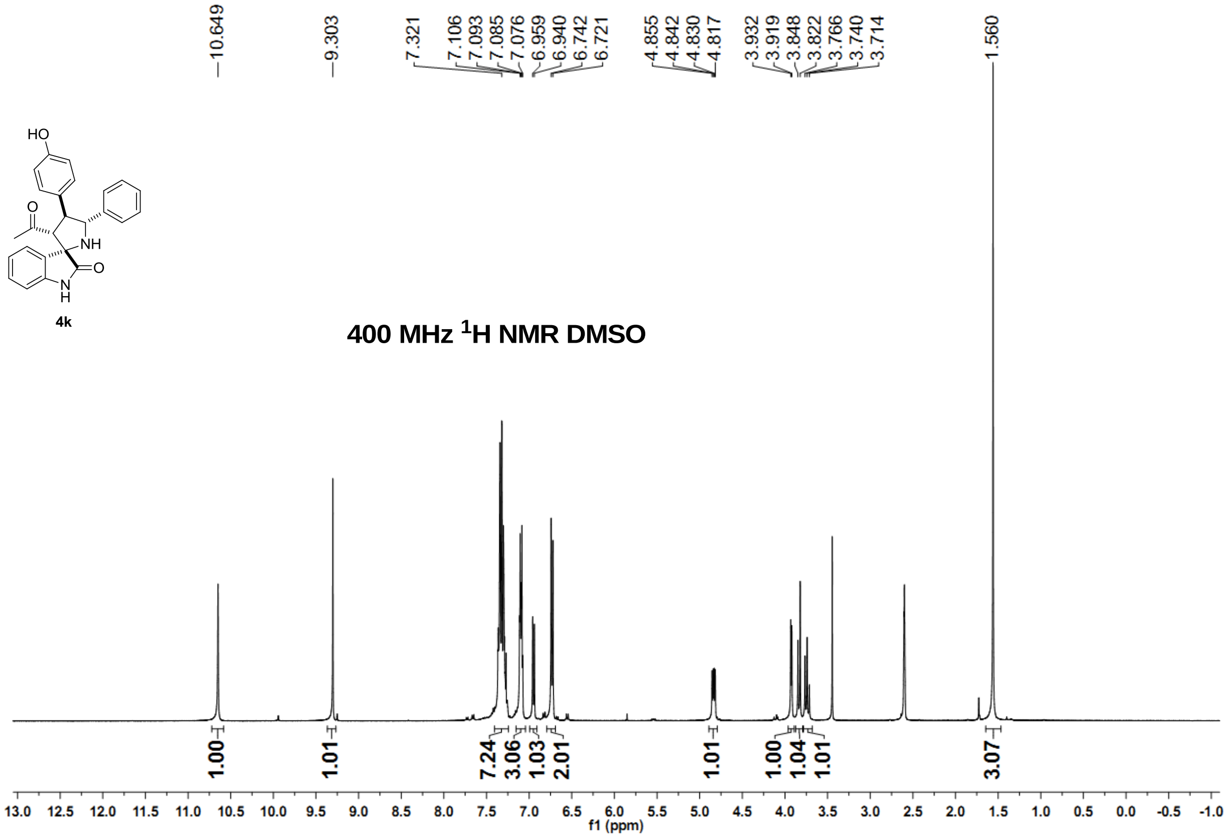
HO
O
NH
O
N
H
4k
400 MHz 1H NMR DMSO
10.649
9.303
7.321
7.106
7.093
7.085
7.076
6.959
6.940
6.742
6.721
4.855
4.842
4.830
4.817
3.932
3.919
3.848
3.822
3.766
3.740
3.714
1.560
1.00
1.01
7.24
3.06
1.03
2.01
1.01
1.00
1.04
1.01
3.07
13.0 12.5 12.0 11.5 11.0 10.5 10.0 9.5 9.0 8.5 8.0 7.5 7.0 6.5 6.0 5.5 5.0 4.5 4.0 3.5 3.0 2.5 2.0 1.5 1.0 0.5 0.0 -0.5 -1.0
f1 (ppm)

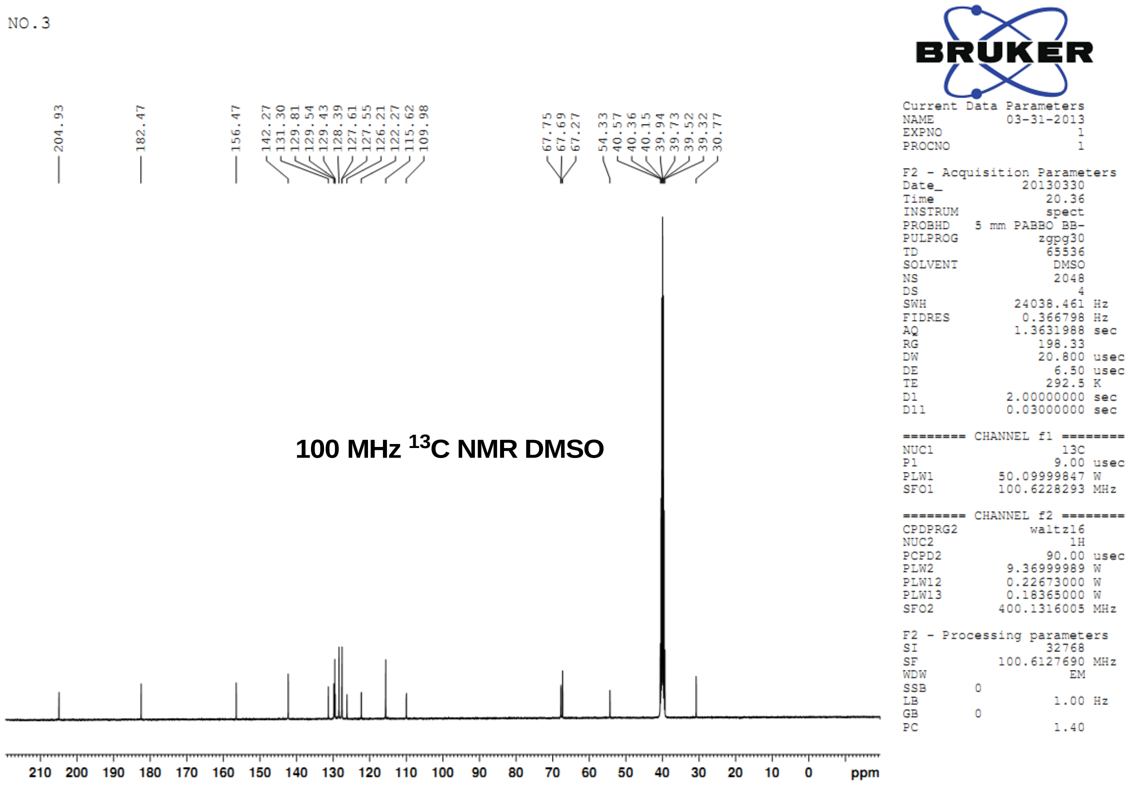
NO.3
204.93
182.47
156.47
142.27
131.30
129.81
129.54
129.43
128.39
127.61
127.55
126.21
122.27
115.62
109.98
67.75
67.69
67.27
54.33
40.57
40.36
40.15
39.94
39.73
39.52
39.32
30.77
100 MHz 13C NMR DMSO
210 200 190 180 170 160 150 140 130 120 110 100 90 80 70 60 50 40 30 20 10 0 ppm
BRUKER
Current Data Parameters
NAME 03-31-2013
EXPNO 1
PROCNO 1
F2 - Acquisition Parameters
Date_ 20130330
Time 20.36
INSTRUM spect
PROBHD 5 mm PABBO BB-
PULPROG zgpg30
TD 65536
SOLVENT DMSO
NS 2048
DS 4
SWH 24038.461 Hz
FIDRES 0.366798 Hz
AQ 1.3631988 sec
RG 198.33
DW 20.800 usec
DE 6.50 usec
TE 292.5 K
D1 2.00000000 sec
D11 0.03000000 sec
======== CHANNEL f1 ========
NUC1 13C
P1 9.00 usec
PLW1 50.09999847 W
SFO1 100.6228293 MHz
======== CHANNEL f2 ========
CPDPRG2 waltz16
NUC2 1H
PCPD2 90.00 usec
PLW2 9.36999989 W
PLW12 0.22673000 W
PLW13 0.18365000 W
SFO2 400.1316005 MHz
F2 - Processing parameters
SI 32768
SF 100.6127690 MHz
WDW EM
SSB 0
LB 1.00 Hz
GB 0
PC 1.40
